# The global survival rate of graft and patient in kidney transplantation of children: a systematic review and meta-analysis

**DOI:** 10.1186/s12887-022-03545-2

**Published:** 2022-08-24

**Authors:** Mousa Ghelichi-Ghojogh, Fateme Mohammadizadeh, Fatemeh Jafari, Mouhebat Vali, Sepideh Jahanian, Masoud Mohammadi, Alireza Jafari, Rozhan Khezri, Hossein-Ali Nikbakht, Masumeh Daliri, Abdolhalim Rajabi

**Affiliations:** 1 Health Management and Social Development Research Center, Faculty of Health, Golestan University of Medical Sciences, Gorgan, Iran; 2 Department of Epidemiology, Shiraz University of Medical Sciences, Shiraz, Iran; 3 Department of Cardiovascular Surgery, Mayo Clinic, College of Medicine and Science, Rochester, MN USA; 4 Research Center of Gastroenterology and Hepatology, Golestan University of Medical Sciences, Gorgan, Iran; 5 Department of Health Education and Health Promotion, School of Health, Social Development and Health Promotion Research Center, Gonabad University of Medical Sciences, Gonabad, Iran; 6 Urmia Health center, Urmia University of Medical Sciences, Urmia, Iran; 7 Social Determinants of Health Research Center, Health Research Institute, Babol University of Medical Sciences, Babol, Iran

**Keywords:** Kidney transplantation, Graft survival, Patient survival, Meta-analysis

## Abstract

**Background:**

This study is a systematic review and meta-analysis on published studies about the Global Survival Rate of Graft and Patients in the Kidney Transplantation of children.

**Methods:**

Studies that investigated the survival rate of kidney transplants published until the 30th of December 2020 were selected using a systematic search strategy in the following databases: Medline, Embase, Scopus, ProQuest, ISI Web of Science, and Cochrane. The extracted data were entered into the Excel software and STATA 16.0. The search identified 6007 study references. From the total, we excluded 1348 duplicates, 3688 reference titles and abstracts that were deemed irrelevant, and 846 references that were not original articles (i.e., letter, commentary, review) or did not meet the inclusion criteria. As such, 89 studies involving 12,330 participants were included in this meta-analysis.

**Results:**

In this study 1, 3, 5, 7 and 10-year survival rates of graft were estimated to be 92, 83, 74.40, 67.10, and 63.50%, respectively. Also, 1, 3, 5, 7 and 10-year survival rates of patients were estimated to be 99.60, 97.30, 95.20, 74.60, and 97.90%, respectively.

**Conclusions:**

The findings suggest differences in graft and patient survival among children with kidney transplants. Although differences in ethnic origin, incompatibility with deceased donor kidneys, and types of kidney disease are unavoidable, interventions to improve preventive and living-donor transplantation are particularly needed in minority groups. In addition, more research is needed to establish and address the contribution of medical and sociocultural barriers to preferential treatment of these groups.

**Supplementary Information:**

The online version contains supplementary material available at 10.1186/s12887-022-03545-2.

## Background

The prevalence of ESRD (End Stage Renal Disease) is currently increasing as a global health problem in both developed and developing countries [1–3]. Chronic kidney disease (CKD) is a disease associated with irreversible kidney damage that can progress to ESRD (end-stage renal disease) [4–8]. ESRD is a destructive disorder that is associated with extreme mortality and cardiovascular complications, and certain problems such as developmental and psychosocial disorders occur in children, all of which severely affect quality of life [9, 10]. Chronic kidney disease (CKD) is a public health problem that affects the general population and has significantly grown in recent years. Kidney transplantation is the treatment of choice for children with ESRD [11, 12]. Transplantation also improves survival, growth, and health-related quality of life compared to dialysis. Due to new immunosuppressant drugs, the incidence of transplant rejection has decreased, and transplant success has improved. Since children receiving a kidney transplant have a longer life expectancy, it is important to maximize the transplant function and survival of the transplant in this population [13–15]. In the literature, the 5-year survival of transplantation is 85% from living donors and 78% from deceased donors. Overall survival is steadily improving over time [16]. The most common causes of ESRD in children are congenital, cystic, and inherited diseases, while primary and secondary glomerular diseases are the major cause in adolescents [17]. A kidney transplant is the transfer of a healthy kidney from a compatible donor to the body of another person with a nonfunctional kidney and is the most promising option for patients with ESRD. The first organ transplant was performed in Boston, the USA, on 23/Dec/1954 with a living donor, and the first kidney transplant in the Middle East was performed in Shiraz, Iran, in 1967. The main sources of kidney transplantation are live donors (related to consanguinity or affinity), unrelated for financial benefit or altruistic motivation [15, 18]. CKD in children presents with clinical characteristics specific to their age, such as the impact on growth. Also, some of the typical characteristics of pediatric CKD, such as the etiology and cardiovascular complications, represent variables that not only influence the patient’s health during childhood but also impact their future adult life. Moreover, CKD has an important psychosocial impact on the patient and the immediate family [19, 20]. Studies show that kidney transplantation improves survival, life expectancy, and quality of life compared to dialysis treatment for ESRD patients. The present study is a systematic review and meta-analysis on the published studies to determine the Global Survival Rate of Graft and Patient in Kidney Transplantation of children.

## Methods

### Search strategy

A comprehensive search was done systematically through PubMed, Scopus, Web of Science, ProQuest, and Cochrane published documents up to the 30th of December 2020. Free text words and medical subject heading (MeSH) terms were used. Details of the search strategy have been provided in Table S3.

### Selection criteria and methods

Two researcehr evaluating the citations and selected studies. After removing the duplicates, a screening of titles and abstracts was performed. Then, a review of the full text. Any discrepancies related to the inclusion of studies were resolved through detailed discussion between this study’s authors. Only those studies that adequately met the inclusion criteria were selected for meta-analysis. The bibliographic list of the identified studies, and relevant reviews on the subject, were examined for additional material. For a study to be included in the meta-analysis, it should have reported either patient or graft survival rates in subjects. Retrospective studies or studies using a cohort approach to follow-up of kidney transplant recipients were included in the meta-analysis. Case reports or review articles were excluded. Studies that did not report any results of interest or sample size were excluded [21, 22].

### Data extraction and quality assessment

Extraction of relevant data from the included studies was done independently by two authors using a data extraction sheet. The following data from the eligible studies were extracted: the surname of the first author, the year in which the study was published, the geographical location where the study was done, the sample size, the study’s design, and the survival rates. The Newcastle-Ottawa Quality Assessment Scale adapted for observational studies was used for quality assessment of included studies [23].

### Statistical analysis

Statistical analysis was done using STATA version 16.0 (StataCorp, College Station, TX, USA). The outcomes considered were patient survival rates and graft survival rates. For the patient and graft survival outcomes, the reported survival rates were analyzed as follows: (1) survival rate at 1 year of transplant; (2) survival rate at 3-years; (3) survival rate at 5-years; (4) survival rate at 7-years and (5) survival rate at 10- years. All estimates were reported within a 95% confidence interval (CI). The heterogeneity of effects was assessed and quantified by I^2^. Subgroup analysis was done based on the geographical location. To assess the differences in the accumulation of evidence for survival rates, cumulative meta-analyses were conducted [22]. Publication bias was not assessed with funnel plot because the probability of survival is always a positive number between zero and one, and cannot be negative; therefore, all studies were distributed on the right side of the vertical line, and this leads to asymmetry in the funnel plot which is not related to publication bias. Therefore, publication bias was only assessed using Begg’s test.

## Results

### Description of studies

**Fig. 1:**
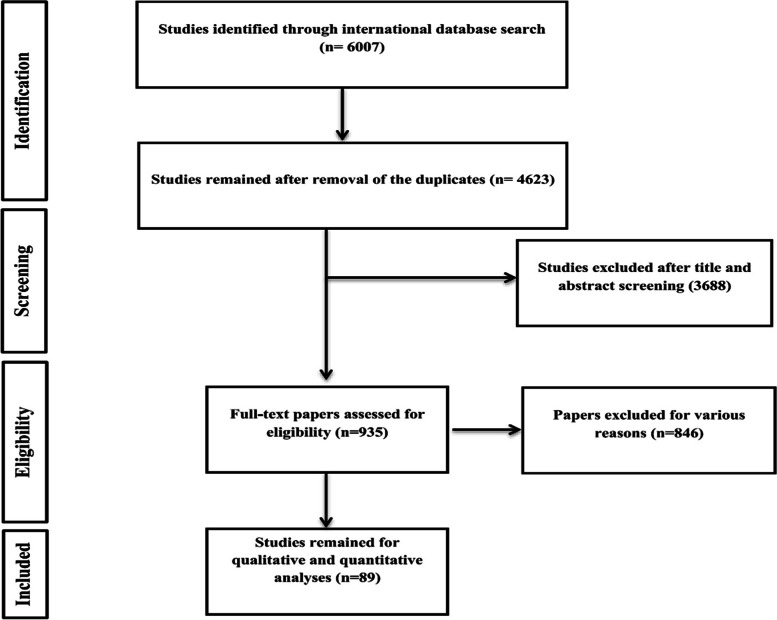
Flowchart of the included eligible studies in systematic review

The search identified 6007 study references. From the total, we excluded 1348 duplicates, 3688 reference titles and abstracts deemed irrelevant, and 846 references that were not original articles (i.e., letter, commentary, review) or did not meet the inclusion criteria. As such, 89 studies involving 12,330 participants were included in this meta-analysis (Fig. 1).

The characteristics of the included studies are listed in Table S1. All studies had a cohort design. Of the 89 studies included in this meta-analysis, 76 studies reported 1-year survival of graft, 31 studies reported 3-year survival of graft, 71 studies reported 5-year survival of graft, 11 studies reported 7-year survival of graft, and 40 studies reported 10-year survival of the graft. Also, of the 89 studies included in this meta-analysis, 50 studies reported 1-year survival of kidney transplant patients, 23 studies reported 3-year survival of kidney transplant patients, 53 studies reported 5-year survival of kidney transplant patients, 4 studies reported 7-year survival of kidney transplant patients, and 30 studies reported 10-year survival of kidney transplant patients. Based on geographical location, 37 studies were from Asia, 32 from Europe, 15 from North America, 4 from South America, and 1 from North Africa. All the included studies were non-randomized, and most were retrospective medical record-based.

### Quality assessment

Overall, 54 studies had a good quality, and 35 of them had fair quality. Table S2 presents the findings of the quality assessment of included studies.

### Pooled evidence for graft survival

#### Pooled 1-year survival rate of graft

**Fig. 2:**
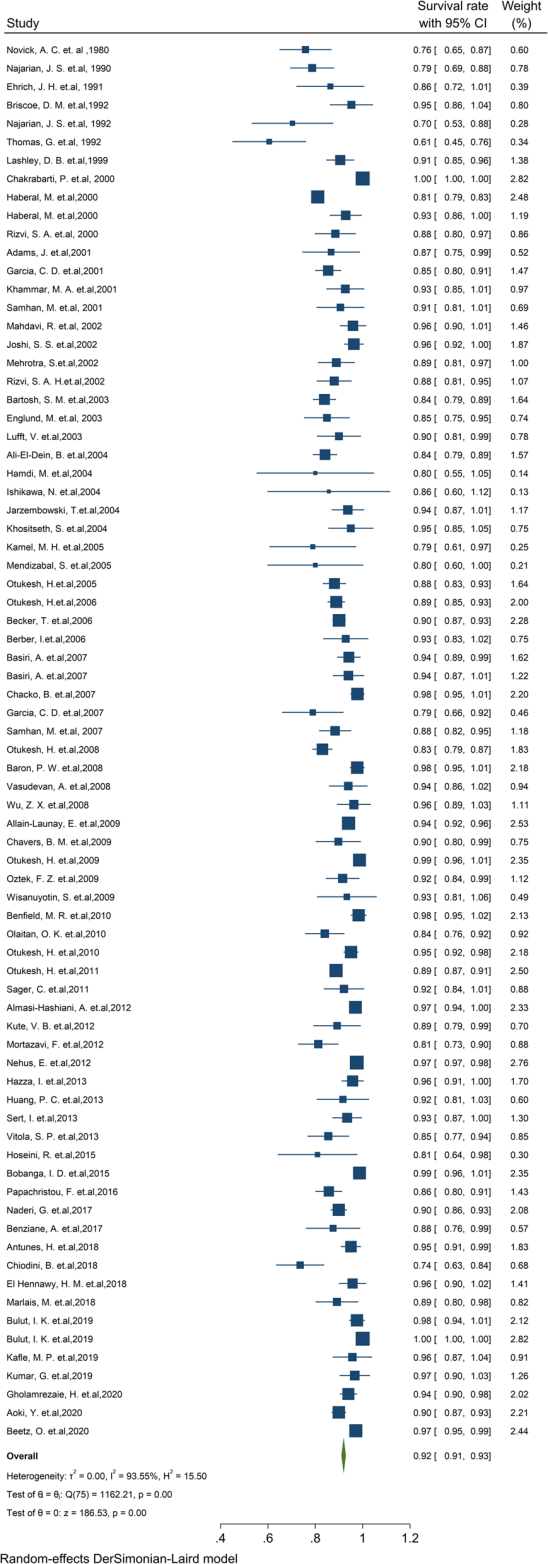
One-year survival rate of Graft kidney transplantation in children

Of the final articles, 76 studies illustrated 1-year survival rate of the graft kidney. Based on the random-effect model, the study demonstrated that 1-year survival rates of the graft kidney were 92% (95% CI, 91.0–92.9) (Fig. 2). There was no evidence of publication bias (Begg’s *P* = 0.64). Furthermore, the 1-year survival by continents in descending order is as follows: Asia 92.1% (95% CI: 90.4–93.7), North America 91.1% (95% CI: 87.2–95), Europe 90.5% (95% CI: 87.9–93.1), North Africa 87.5% (95% CI: 76–99) and South America 86.2% (95% CI: 82.2–90.3) (Table 1). The cumulative meta-analysis of 1-year survival was also presented in Fig. S1. The results showed that with an increasing number of studies in later years, graft survival rate is somewhat increased.

**Table 1:** Findings of the subgroup analysis for patient and graft survival

N (no. of studies); pooled effect size (proportion) with 95% C					
	North Africa	North America	Asia	Europe	South America
**Graft Survival**					
1-Year	*N* = 1 87.5% (76–99)	*N* = 12 91.1% (87.2–95),	*N* = 35 92.1% (90.4–93.7)	*N* = 24 90.5% (87.9–93.1)	*N* = 4 86.2% (82.2–90.3)
3-Year	*N* = 0 -	*N* = 8 80.7% (72.8–88.6)	*N* = 16 83.9% (80.1–87.8)	*N* = 7 82.1% (73.4–90.8)	*N* = 0 -
5-Year	*N* = 0 -	*N* = 11 72.2% (64.8–79.5)	*N* = 30 80.8% (77.8–83.9)	*N* = 26 78.5% (75–82)	*N* = 4 73.3% (62.8–83.9)
7-Year	*N* = 1 65.6% (49.2–82.1)	*N* = 1 58.7% (47.5–69.8)	*N* = 9 68.1% (59.7–76.6)	*N* = 0 -	*N* = 0 -
10-Year	*N* = 0 -	*N* = 4 58.1% (41.7–74.5)	*N* = 16 64.3% (57.5–71.1)	*N* = 20 64.2% (58.9–69.5)	*N* = 0 -
**Patient Survival**					
1-Year	*N* = 1 96.9% (90.8–1.00)	*N* = 11 99.9% (99.7–1.00)	*N* = 19 99.9% (99.7–1.00)	*N* = 16 97.1% (95.8–98.4)	*N* = 3 94.2% (91.3–97)
3-Year	*N* = 0 -	*N* = 9 99.3% (98.5–1.00)	*N* = 8 89.3% (85.2–93.4)	*N* = 6 96.3% (92.7–99.8)	*N* = 0 -
5-Year	*N* = 0 -	*N* = 12 97.5% (96.5–98.5)	*N* = 19 94.9% (93.8–96.1)	*N* = 19 93.9% (92.1–95.6)	*N* = 3 90.5% (83.5–97.5)
7-Year	*N* = 1 84.4% (71.8–97.0)	*N* = 1 77.3% (67.9–86.8)	*N* = 2 70.3% (64.4–76.2)	*N* = 0 -	*N* = 0 -
10-Year	*N* = 0 -	*N* = 6 88.8% (80.8–96.9)	*N* = 9 79.1% (71.2–87.0)	*N* = 15 91.9% (89.9–94.0)	*N* = 0 -

#### Pooled 3-year survival rate of graft

**Fig. 3:**
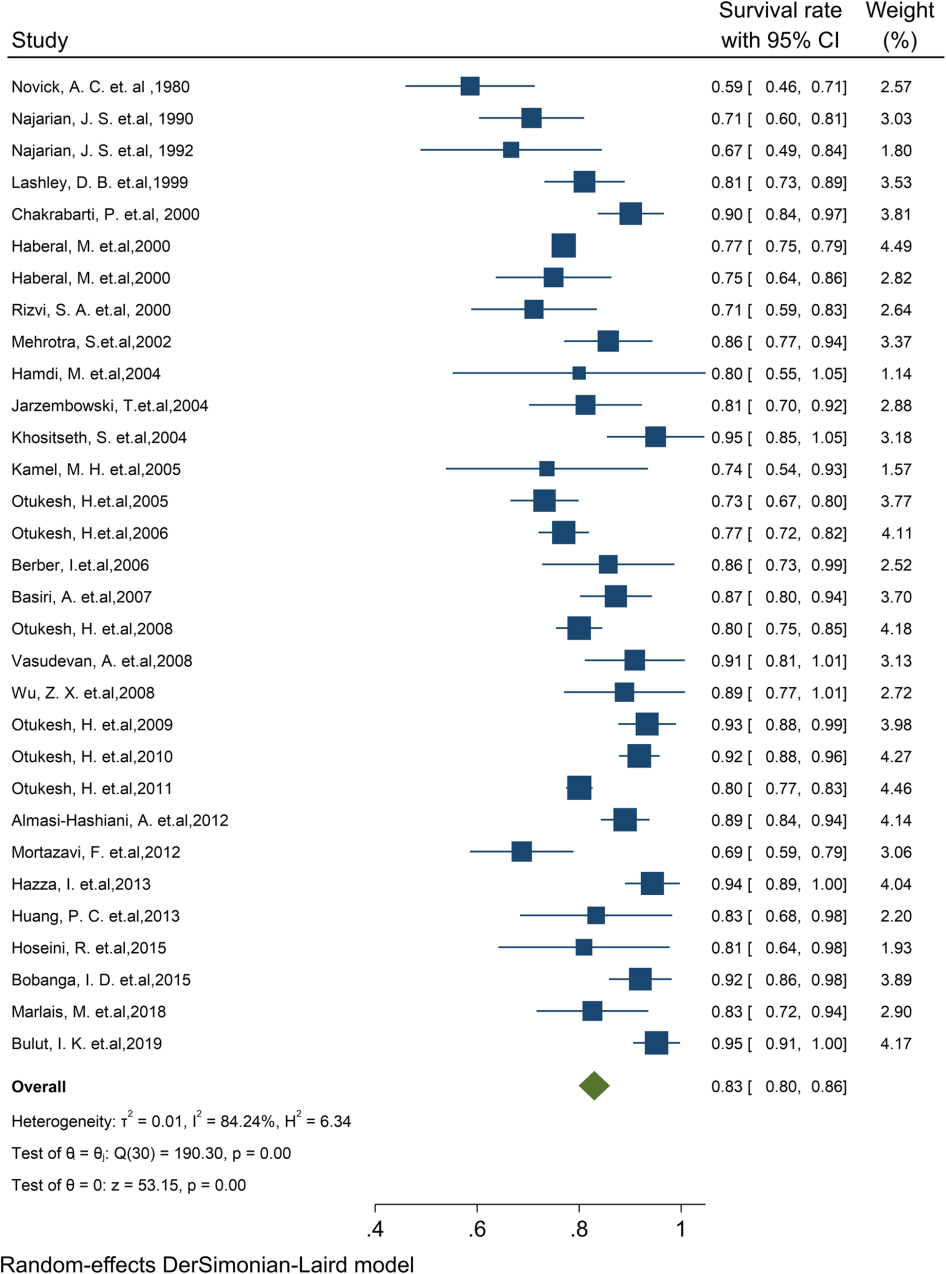
Three-year survival rate of Graft kidney transplantation in children

Of the final articles, 31 studies illustrated the 3-year survival rate of the graft kidney. Based on the random-effect model, the study demonstrated that 3-year survival rates of the graft kidney were 83% (95% CI, 79.9–86.0) (Fig. 3). There was no evidence of publication bias (Begg’s *P* = 0.99). Furthermore, the 3-year survival by continent in descending order was: Asia 83.9% (95% CI: 80.1–87.8), Europe 82.1% (95% CI: 73.4–90.8), and North America 80.7% (95% CI: 72.8–88.6) (Table 1). The cumulative meta-analysis of 3-year survival was also presented in Fig. S2. The results showed that with an increasing number of studies in later years, graft survival rate is considerably increased.

#### Pooled 5-year survival rate of graft

**Fig. 4:**
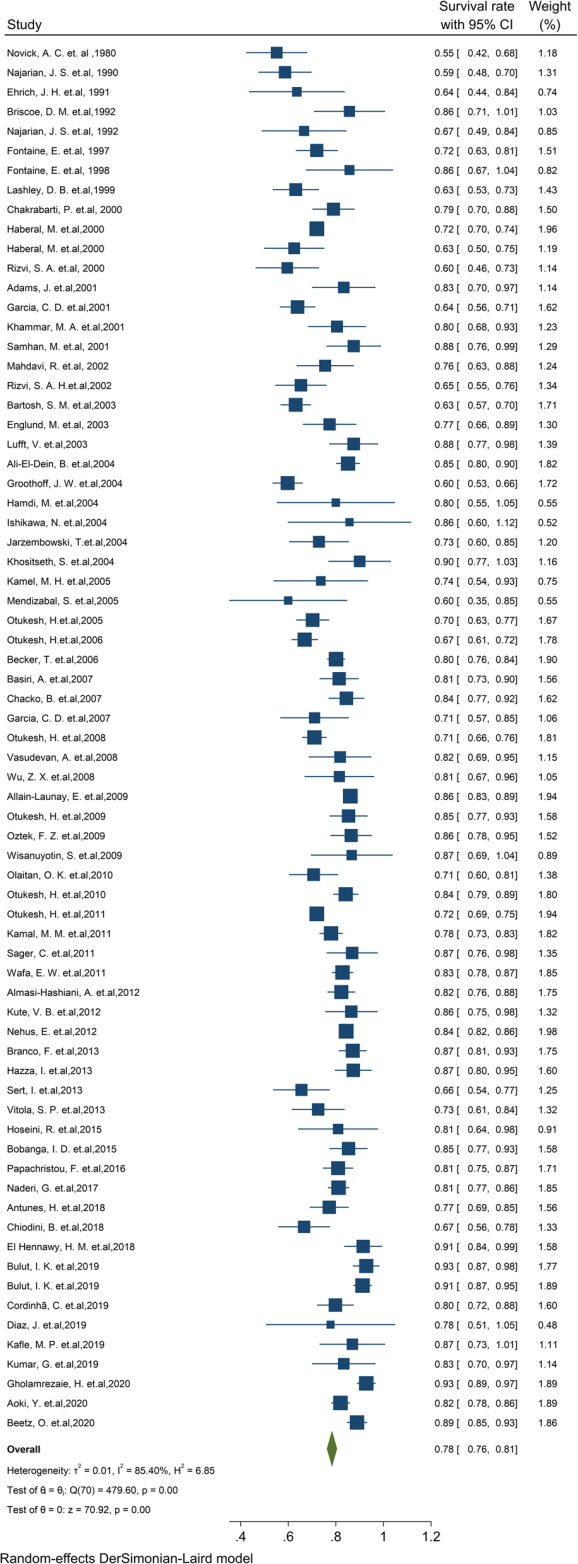
Five-year survival rate of Graft kidney transplantation in children

Of the final articles, 71 studies illustrated the 5-year survival rate of the graft kidney. Based on the random-effect model, the study demonstrated that 5-year survival rates of the graft kidney were 78.4% (95% CI, 76.2–80.5) (Fig. 4). There was no evidence of publication bias (Begg’s *P* = 0.99). Furthermore, the 5-year survival by continents in descending order was: Asia 80.8% (95% CI: 77.8–83.9), Europe 78.5% (95% CI: 75–82), South America 73.3% (95% CI: 62.8–83.9) and North America 72.2% (95% CI: 64.8–79.5) (Table 1). The cumulative meta-analysis of 5-year survival was also presented in Fig. S3. The results showed that with an increasing number of studies in later years, graft survival rate is considerably increased.

#### Pooled 7-year survival rate of graft

Of the final articles, 11 studies illustrated the 7-year survival rate of the graft kidney. Based on the random-effect model, the study demonstrated that 7-year survival rates of the graft kidney were 67.1% (95% CI: 59.6–74.6). There was no evidence of publication bias (Begg’s *P* = 0.81). Furthermore, the 7-year survival by continents in descending order was: Asia 68.1% (95% CI:59.7–76.6), North Africa 65.6% (95% CI: 49.2–82.1) and North America 58.7% (95% CI: 47.5–69.8) (Table 1). Based on a cumulative meta-analysis of 7-year survival, the results showed that with an increasing number of studies in later years, the graft survival rate is somewhat increased.

#### Pooled 10-year survival rate of graft

**Fig. 5:**
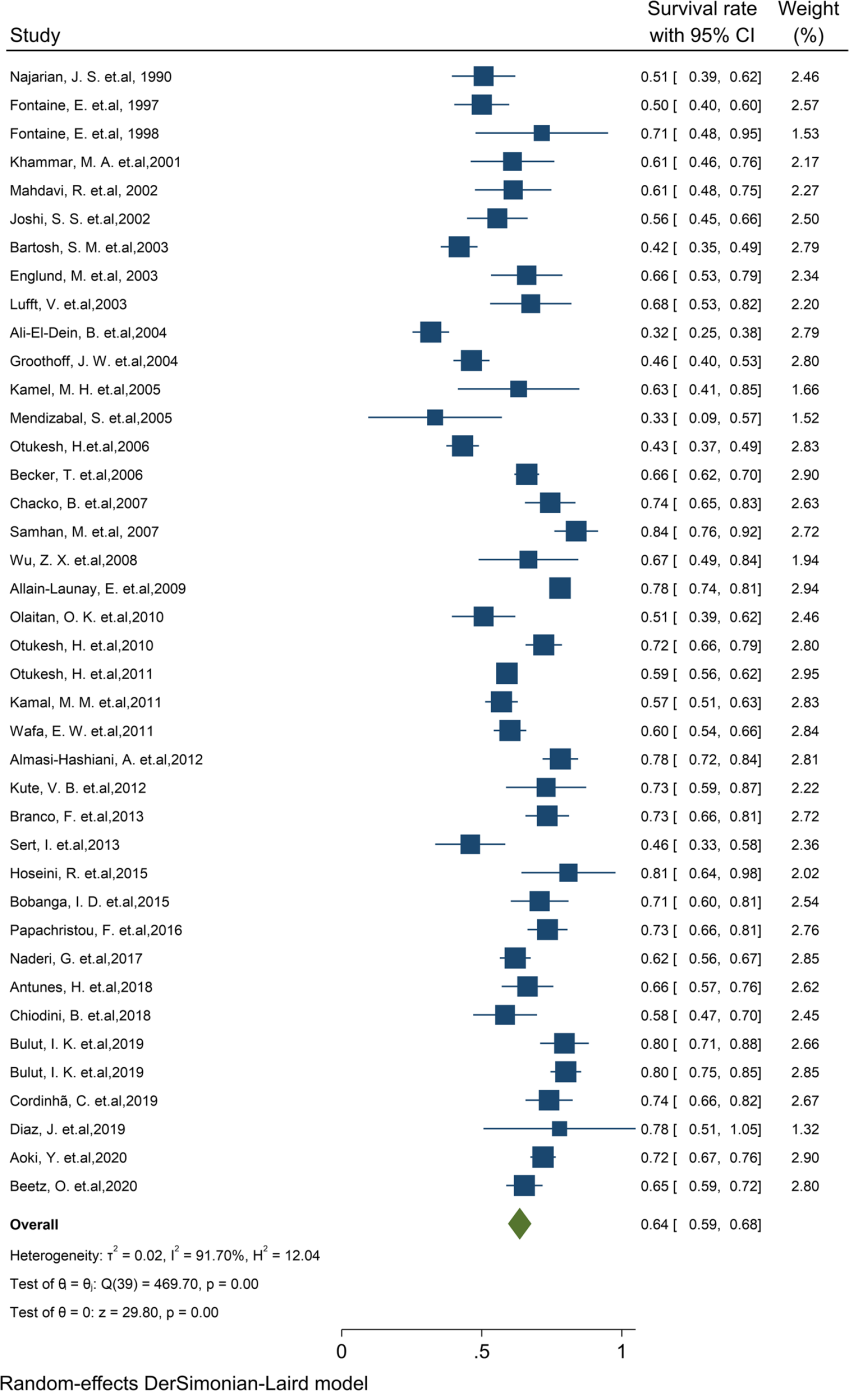
Ten-year survival rate of Graft kidney transplantation in children

Of the final articles, 40 studies illustrated the 10-year survival rate of the graft kidney. Based on the random-effect model, the study demonstrated that 10-year survival rates of the graft kidney were 63.5% (95% CI: 59.4–67.7) (Fig. 5). There was no evidence of publication bias (Begg’s *P* = 0.90). Furthermore, the 10-year survival by continents in descending order was: Asia 64.3% (95% CI: 57.5–71.1), Europe 64.2% (95% CI: 58.9–69.5) and North America 58.1% (95% CI: 41.7–74.5) (Table 1). The cumulative meta-analysis of 10-year survival was also presented in Fig. S4. The results showed that the graft survival rate somewhat increased with an increasing number of studies in later years.

### Pooled evidence for patients survival

#### Pooled 1-year survival rate of patients

**Fig. 6:**
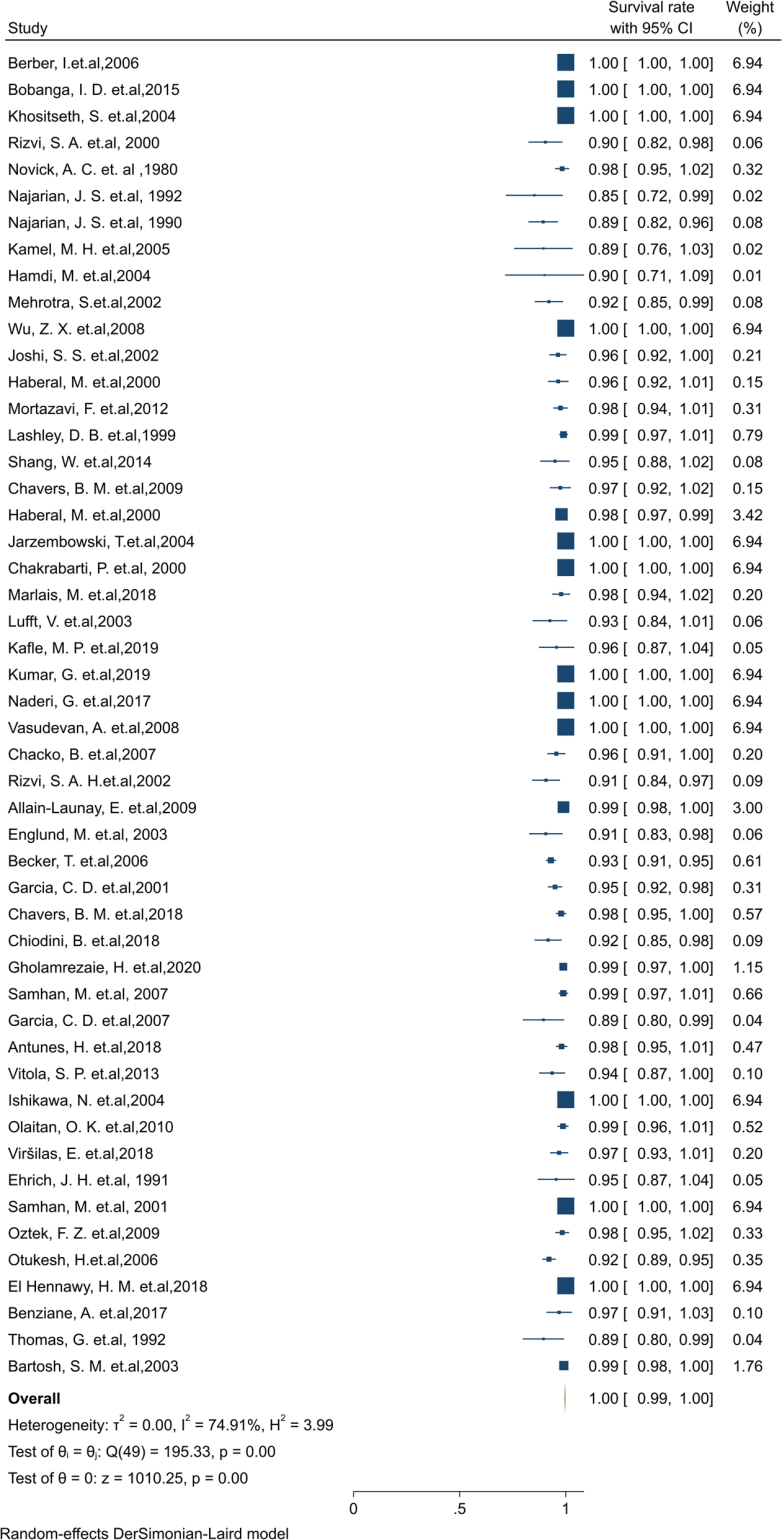
One-year survival rate of Patients kidney transplantation in children

Of the final articles, 50 studies illustrated 1-year survival rate of patients. Based on the random-effect model, the overall pooled patient survival rate at a 1-year post-transplantation period was 99.6% (95% CI: 99.4–99.8) (Fig. 6). There was no evidence of publication bias (Begg’s *P* = 0.99). Furthermore, the 1-year survival by continents in descending order was: Asia 99.9% (95% CI:99.7–1.00), North America 99.9% (95% CI: 99.7–1.00), Europe 97.1% (95% CI: 95.8–98.4), North Africa 96.9% (95% CI: 90.8–1.00) and South America 94.2% (95% CI: 91.3–97) (Table 1). The cumulative meta-analysis of 1-year survival was also presented in Fig. S5. The results showed that with an increasing number of studies in later years, the survival rate of patients is somewhat increased.

#### Pooled 3-year survival rate of patients

**Fig. 7:**
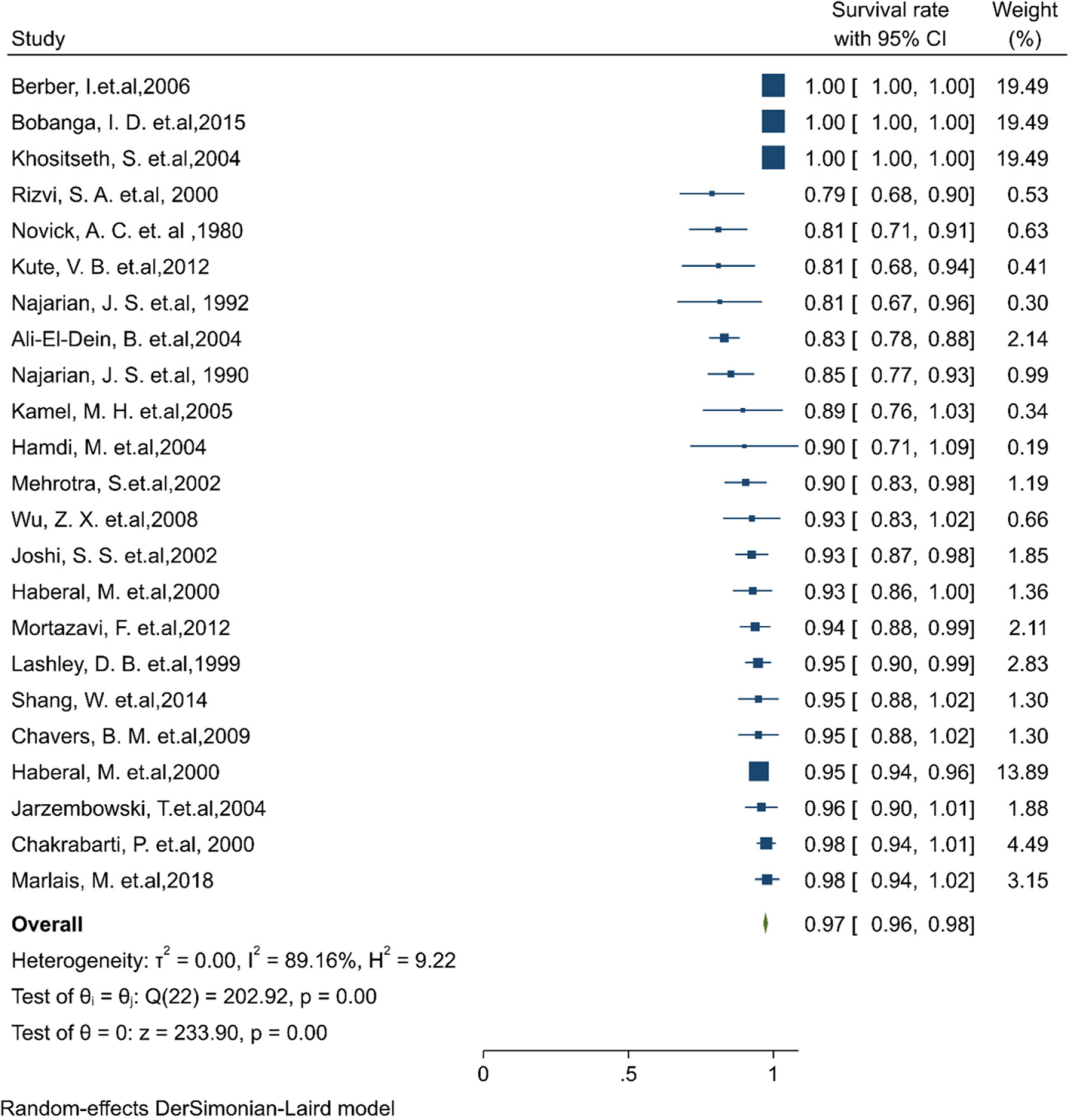
Three-year survival rate of Patients kidney transplantation in children

Of the final articles, 23 studies illustrated the 3-year survival rate of patients. Based on the random-effect model, the overall pooled patient survival rate at the 3-year post-transplantation period was 97.3% (95% CI: 96.5–98.1) (Fig. 7). There was no evidence of publication bias (Begg’s *P* = 0.99). Furthermore, the 3-year survival by continents in descending order was: North America 99.3% (95% CI: 98.5–1.00), and Europe 96.3% (95% CI: 92.7–99.8), and Asia 89.3% (95% CI:85.2–93.4) (Table 1). The cumulative meta-analysis of 3-year survival was also presented in Fig. S6. The results showed that with an increasing number of studies in later years, the survival rate of patients is considerably increased.

#### Pooled 5-year survival rate of patients

**Fig. 8:**
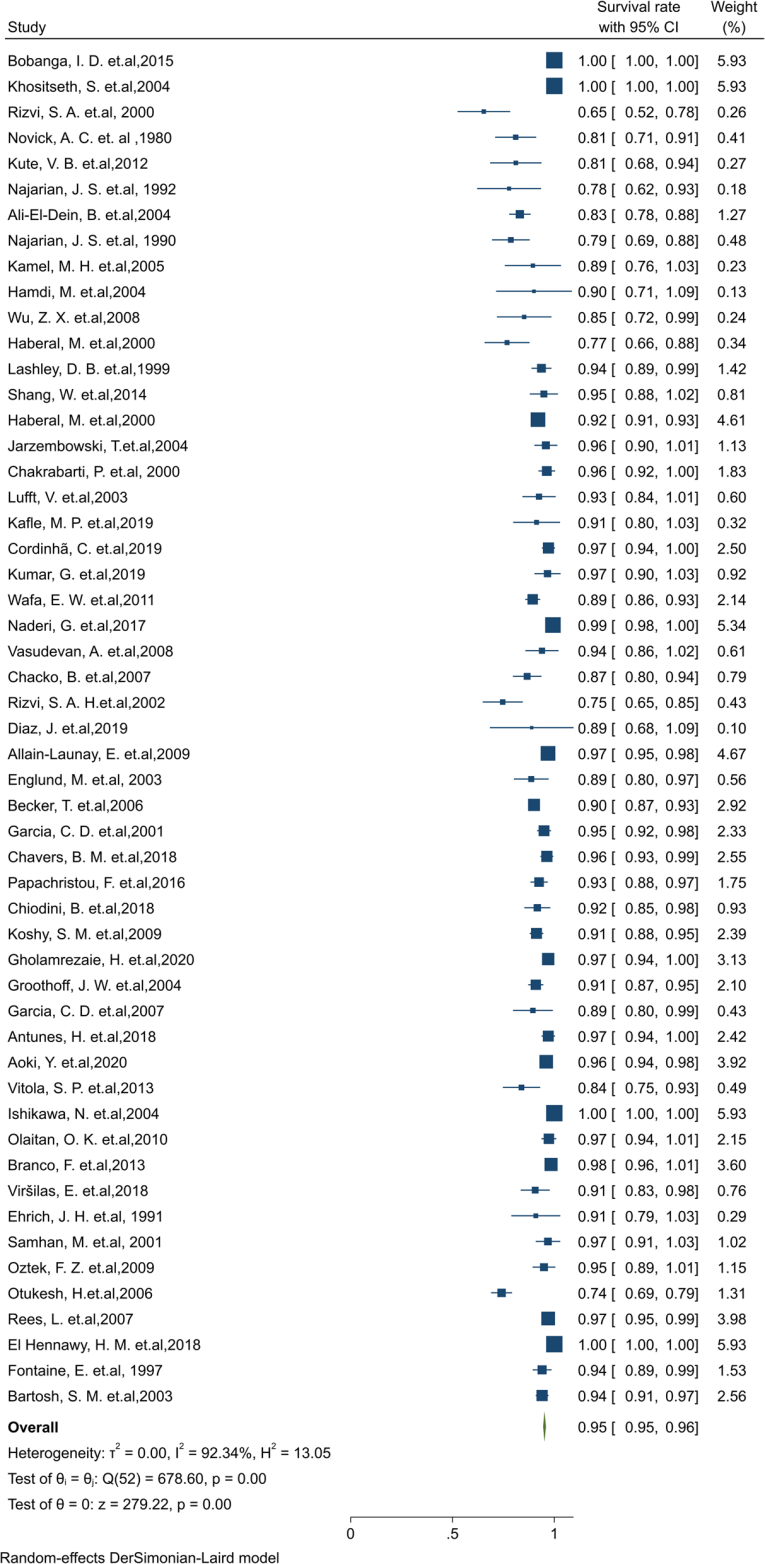
Five-year survival rate of patients kidney transplantation in children

Of the final articles, 53 studies illustrated the 5-year survival rate of patients. Based on the random-effect model, the overall pooled patient survival rate at the 5-year post-transplantation period was 95.2% (95% CI: 94.5–95.9) (Fig. 8). There was no evidence of publication bias (Begg’s *P* = 0.46). Furthermore, the 5-year survival by continents in descending order was: North America 97.5% (95% CI: 96.5–98.5), Asia 94.9% (95% CI:93.8–96.1), Europe 93.9% (95% CI: 92.1–95.6), and South America 90.5% (95% CI: 83.5–97.5) (Table 1). The cumulative meta-analysis of 5-year survival was also presented in Fig. S7. The results showed that with an increasing number of studies in later years, the survival rate of patients is somewhat increased.

#### Pooled 7-year survival rate of patients

Of the final articles, 4 studies illustrated the 7-year survival rate of patients. Based on the random-effect model, the overall pooled patient survival rate at the 7-year post-transplantation period was 74.6% (95% CI: 68.3–81.0). There was no evidence of publication bias (Begg’s *P* = 0.73). Furthermore, the 7-year survival by continents in descending orders was: North Africa 84.4% (95% CI: 71.8–97.0), North America 77.3% (95% CI: 67.9–86.8) and Asia 70.3% (95% CI: 64.4–76.2) (Table 1). Based on a cumulative meta-analysis of 7-year survival, the results showed that the survival rate of patients is unchanged with an increasing number of studies in later years.

#### Pooled 10-year survival rate of patients

**Fig. 9:**
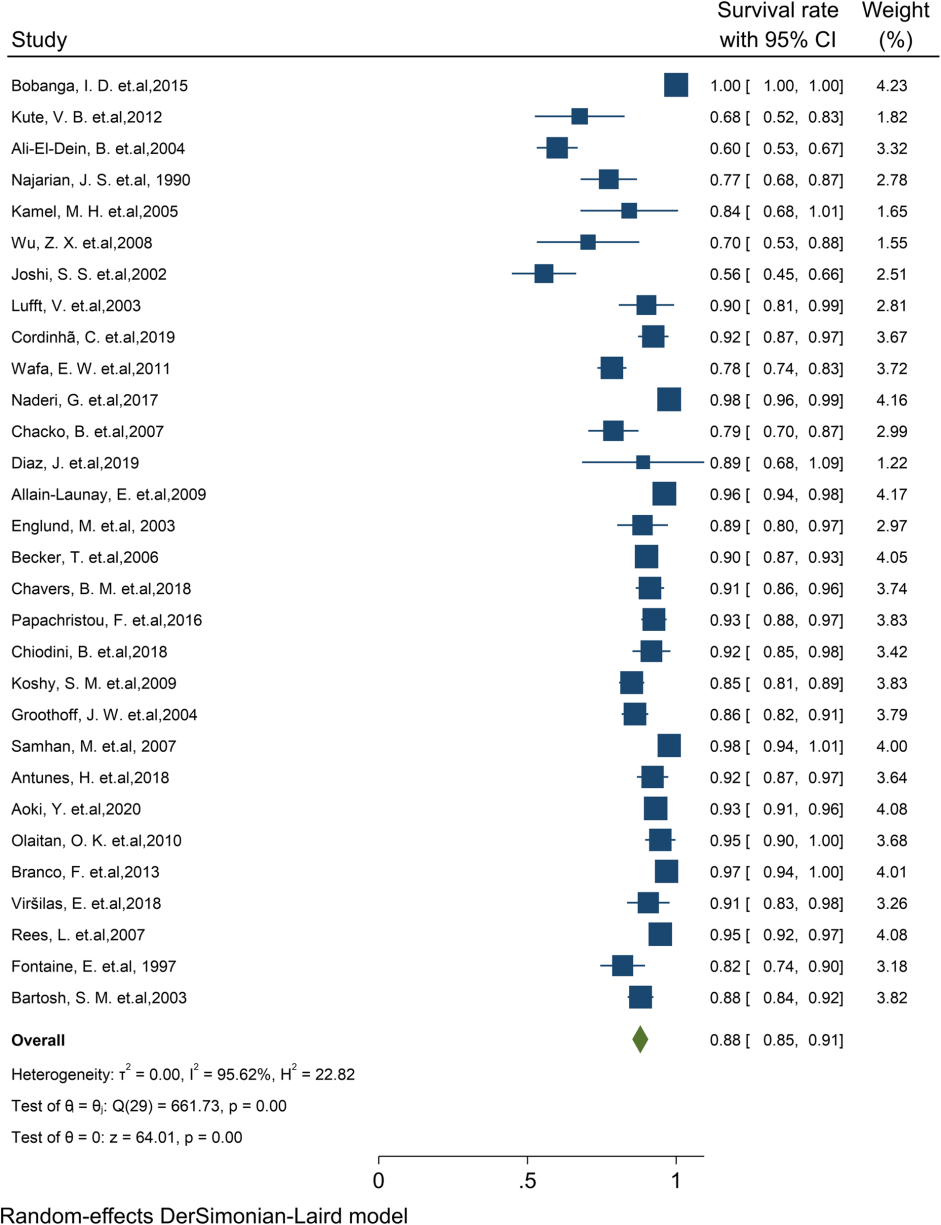
Ten-year survival rate of Patients kidney transplantation in children

Of the final articles, 30 studies illustrated the 10-year survival rate of patients. Based on the random-effect model, the overall pooled patient survival rate at the 10-year post-transplantation period was 87.9% (95% CI: 85.2–90.6) (Fig. 9). There was no evidence of publication bias (Begg’s *P* = 0.88). Furthermore, the 10-year survival by continents in descending order was: Europe 91.9% (95% CI: 89.9–94.0), North America 88.8% (95% CI: 80.8–96.9), and Asia 79.1% (95% CI: 71.2–87.0) (Table 1). Based on a cumulative meta-analysis of 10-year survival, the results showed that the survival rate of patients is considerably increased with an increasing number of studies in later years.

## Discussion

In this study, we investigated the survival of children and grafts after kidney transplantation. The results of our meta-analysis study show that the survival of 1-year, 3-year, 5-year, 7-year and 10-year transplants are 92, 83, 78.4, 67.1%, and 63.5, respectively. In all, one-, three-, five-, seven-, and 10-year graft survival is higher in Asia than on any other continent. North America has the lowest graft survival, except for 1-year of graft survival, whereas South America reports lower.

Survival rates of 1, 3, 5, 7 and 10 years after transplantation are 99.6, 97.3, 95.2, 74.6, and 87.9%, respectively. Except for the 1-year survival of patients, which is higher in Asia, the rest of the endpoints have a lower survival rate, which contrasts with the positive graft survival that seems favorable in Asia.

This study of children with ESRD showed that transplant survival and patient survival were not significantly different in several geographical areas. In a study by Lidwien A. Tjaden et al. [24], Transplant survival rates were similar in different racial groups, but in the study, Asian patients were 2.5 times more likely to die than white patients, which differed from the results of our study. This was partly explained by differences in the initial distribution of kidney disease. However, another study suggests that differences in survival in kidney patients may be explained by racial differences [24] .

Studies suggest that black and Hispanic patients have less access to kidney transplantation than white people, even after adjusting for the individual-level and neighborhood-level measures of socioeconomic status [24–28]. These differences have been observed even in countries with universal access to health care, such as Indigenous children in Canada, Australia, New Zealand [19, 20, 23], migrant children, adolescents, and young people in the Netherlands and Belgium [29–32].

The causes of differences in kidney transplantation in children are thought to be multifactorial and may be influenced by a complex mix of biological, socioeconomic, and cultural factors. Given the minor differences in geographical areas, one of the hypotheses could be that primary kidney disease is significantly higher in areas with lower survival, which carries a considerable risk of disease recurrence and subsequent transplant loss. This increased incidence of ESRD explains, to some extent, the difference in access to transplantation [24, 33–36]. However, we did not have the category of primary renal disease information available to assess graft access accurately. For this reason, only the difference in graft access does not fully explain all the variations of survival in different geographical areas, and extra non-biological factors are also possibly involved.

In addition, part of the difference may be due to patients’ varied opinions about the transplant. A Dutch study looking to accept live donor kidney transplants among patients with ESRD recently found other non-medical factors that led to reduced living-related donation and thus longer dialysis time among groups identified as a minority. These factors include different beliefs and attitudes toward donating due to insufficient knowledge and communication in their social network and misunderstandings and miscommunication between patients and their families [37–40]. Behavioral considerations may point towards differences in access to kidney transplantation and patients’ sociodemographic and educational status [37, 41–43]. The patients’ racial background may also influence the physician’s views on the patient’s behavior and the likelihood of successful treatment [44–48].

Our study has several strengths, including comparisons between different continents and reporting both graft survival and patient survival at 1-year, 3-year, 5-year, 7-year, and 10-year. However, there are limitations. First, we did not have detailed information on patients from minority groups and races, so it is suggested that data collection on the racial background and socioeconomic status should be strongly encouraged in the records to investigate identified differences further. Also, we did not have comprehensive clinical information and socioeconomic status data for a sufficient number of patients. As a result, we could not separate biological effects other than primary kidney disease (e.g., blood type and genotype) from socioeconomic and racial backgrounds.

## Conclusion

In conclusion, The findings suggest differences in graft and patient survival among children with kidney transplants. Perhaps well-known biological aspects related to racial backgrounds, such as primary kidney disease, can only partially explain this difference, and other biological and social aspects of the environment may be involved. Complex interactions between biological and social environments require further study to guide targeted interventions to reduce this disparity across racial subgroups. Although differences in ethnic origin, incompatibility with deceased donor kidneys, and types of kidney disease are unavoidable, interventions to improve preventive and living-donor transplantation are particularly needed in minority groups. In addition, more research is needed to identify and address the contribution of medical and socio-cultural barriers to preferential treatment among specific groups.

## Supplementary Information

**Additional file 1.**

**Additional file 2.**

**Additional file 3.**

**Additional file 4.**

## Data Availability

The data generated or analyzed during this study is available from the corresponding author on reasonable request.

## References

[1] Shahbazi F, Ranjbaran M, Karami-far S, Soori H, Jafarimanesh H (2015). Graft survival rate of renal transplantation during a period of 10 years in Iran. J Res Med Sci.

[2] Ghojogh MG, Salarilak S, Afshari AT, Khalkhali HR, Mohammadi-Fallah MR, Makhdoomi K (2018). Impact of type of donor on graft and patient survival rate in kidney transplanted patients in Iran. J Renal Inj Prev.

[3] Ghojogh MG, Salarilak S, Afshari AT, Khalkhali HR, Mohammadi-Fallah MR, Makhdoomi K (2018). The effect of urinary tract infection on patient and graft survival rate in a group of kidney transplanted patients. J Renal Inj Prev.

[4] Zeba Z, Fatema K, Sumit AF, Zinnat R, Ali L. Early screening of chronic kidney disease patients among the asymptomatic adult population in Bangladesh J Prevent Epidemiol. 2020;5(1):e10-e.

[5] Ghelichi-Ghojogh M, Ghaem H, Mohammadizadeh F, Vali M, Ahmed F, Hassanipour S (2021). Graft and patient survival rates in kidney transplantation, and their associated factors: a systematic review and Meta-analysis. Iran J Public Health.

[6] Ghojogh MG, Salarilak S, Afshari AT, Khalkhali HR, Mohammadi-Fallah MR, Mkhdoomi K. The effect of body mass index on patient and graft survival rate in kidney transplanted patients in Iran. Nephro-Urol Monthly. 2017;9(4):e14386.

[7] Ghelichi-Ghojogh M, Fararouei M, Seif M, Pakfetrat M (2022). Chronic kidney disease and its health-related factors: a case-control study. BMC Nephrol.

[8] Ghelichi-Ghojogh M, Seif M, Shahryari B, Pakfetrat M. Impact of social and clinical factors on the diagnostic delay of chronic kidney disease: an evaluation study. Int Urol Nephrol. 2022;54(7):1603-1612. 10.1007/s11255-021-03037-9 34713367

[9] Kari J (2012). Epidemiology of chronic kidney disease in children. J Nephropathol.

[10] Aleebrahim-Dehkordi E, Mazaheri E, Roshan B, Lakkakula BV, Hasanpour-Dehkordi A, Khosravian M, et al. Strive for kidney health for everyone during COVID-19; the possible theme for the world kidney day 2021. J Nephropharmacol. 2021;10(2):e12.

[11] Tayebi KH (2012). Short history about renal transplantation program in Iran and the world: special focus on world kidney day. J Nephropathol.

[12] Silva S, Milano C, García G, Abib A, Díaz C, Laham G. Frailty at the beginning of dialysis; is it a prognostic factor? J Renal Endocrinol 2021;7(1):e13-e.

[13] Friedersdorff F, Koch T-M, Banuelos-Marco B, Gonzalez R, Fuller TF, Von Mechow S (2018). Long-term follow-up after paediatric kidney transplantation and influence factors on graft survival: a single-Centre experience of 16 years. Urol Int.

[14] Saeed B (2013). Pediatric versus adult kidney transplantation activity in Arab countries. Saudi J Kidney Dis Transpl.

[15] van Heurn E, de Vries EE (2009). Kidney transplantation and donation in children. Pediatr Surg Int.

[16] Smith JM, Martz K, Blydt-Hansen TD (2013). Pediatric kidney transplant practice patterns and outcome benchmarks, 1987-2010: a report of the north American pediatric renal trials and collaborative studies. Pediatr Transpl.

[17] Winterberg PD, Garro R (2019). Long-term outcomes of kidney transplantation in children. Pediatr Clin North Am.

[18] Malekshahi A, MortezaNejad HF, Taromsari MR, Gheshlagh RG, Delpasand K (2020). An evaluation of the current status of kidney transplant in terms of the type of receipt among Iranian patients. Renal Replacement Therapy.

[19] Becherucci F, Roperto RM, Materassi M, Romagnani P (2016). Chronic kidney disease in children. Clin Kidney J.

[20] Ookalkar D, Ookalkar A, Gupta V, Balwani M (2021). Clinical profile of chronic kidney disease of unknown origin in patients of Yavatmal district, Maharashtra, India. J Renal Endocrinol.

[21] Hou Y, Wang X, Yang H, Zhong S (2021). Survival and complication of liver transplantation in infants: a systematic review and meta-analysis. Front Pediatr.

[22] Rajabi A, Sharafi H, Alavian SM (2021). Harm reduction program and hepatitis C prevalence in people who inject drugs (PWID) in Iran: an updated systematic review and cumulative meta-analysis. Harm Reduct J.

[23] Peterson J, Welch V, Losos M, Tugwell P (2011). The Newcastle-Ottawa scale (NOS) for assessing the quality of nonrandomised studies in meta-analyses.

[24] Tjaden LA, Noordzij M, van Stralen KJ, Kuehni CE, Raes A, Cornelissen EA (2016). Racial disparities in access to and outcomes of kidney transplantation in children, adolescents, and young adults: results from the ESPN/ERA-EDTA (European Society of Pediatric Nephrology/European renal association− European Dialysis and transplant association) registry. J Am J Kidney Dis.

[25] Patzer RE, Amaral S, Klein M, Kutner N, Perryman J, Gazmararian J (2012). Racial disparities in pediatric access to kidney transplantation: does socioeconomic status play a role?. J Am J Transpl.

[26] Patzer RE, Sayed BA, Kutner N, McClellan WM, Amaral S (2013). Racial and ethnic differences in pediatric access to preemptive kidney transplantation in the United States. J Am J Transpl.

[27] Patzer RE, Perryman JP, Schrager JD, Pastan S, Amaral S, Gazmararian JA (2012). The role of race and poverty on steps to kidney transplantation in the southeastern United States. Am J Transpl.

[28] Krishna K, Satheesh S, Ramanathan G, Paul SF, Matcha J, Elumalai R. Lack of association between ACE I/D, NOS3 VNTR polymorphisms and drug toxicity of tacrolimus treated post-renal transplantation patients. J Nephropharmacol 2019;9(2):e19-e.

[29] Tromp WF, Cransberg K, van der Lee JH, Bouts AH, Collard L, Van Damme-Lombaerts R (2012). Fewer pre-emptive renal transplantations and more rejections in immigrant children compared to native Dutch and Belgian children. J Nephrol Dialysis Transpl.

[30] Oztek FZ, Ipsiroglu O, Mueller T, Aufricht C (2009). Outcome after renal transplantation in children from native and immigrant families in Austria. Eur J Pediatr.

[31] Schoenmaker NJ, Tromp WF, van der Lee JH, Adams B, Bouts AH, Collard L (2012). Disparities in dialysis treatment and outcomes for Dutch and Belgian children with immigrant parents. Pediatr Nephrol.

[32] Tromp WF, Van der Lee JH, Offringa M, Bouts AHM, Collard L, Cransberg K (2011). Lessons learned from efforts to improve the quality of care in children with end-stage renal disease in the Netherlands and Belgium. Arch Dis Child.

[33] Ku E, McCulloch CE, Grimes BA, Johansen KL (2017). Racial and ethnic disparities in survival of children with ESRD. J Am Soc Nephrol.

[34] Laster M, Soohoo M, Hall C, Streja E, Rhee CM, Ravel VA (2017). Racial–ethnic disparities in mortality and kidney transplant outcomes among pediatric dialysis patients. Pediatr Nephrol.

[35] Drukker A, Feinstein S, Rinat C, Rotem-Braun A, Frishberg Y (2003). Cadaver-donor renal transplantation of children in Israel (1990–2001): racial disparities in health care delivery?. Pediatrics..

[36] Assadi F (2012). The epidemic of pediatric chronic kidney disease: the danger of skepticism. J Nephropathol.

[37] Ismail SY, Luchtenburg AE, Gestel JAK-V, Zuidema WC, Weimar W, Busschbach JJ (2013). Modifiable factors in access to living-donor kidney transplantation among diverse populations. J Transp Secur.

[38] Roodnat JI, Laging M, Massey EK, Kho M, Kal-van Gestel JA, Ijzermans JNM (2012). Accumulation of unfavorable clinical and socioeconomic factors precludes living donor kidney transplantation. Transplantation..

[39] Hart A, Bruin M, Chu S, Matas A, Partin MR, Israni AK (2019). Decision support needs of kidney transplant candidates regarding the deceased donor waiting list: a qualitative study and conceptual framework. Clin Transpl.

[40] Safaei-Asl A, Jilani M, Heydarzadeh A, Maleknejad S (2019). Prognosis of acute kidney injury based on pRIFLE criteria among patients admitted to pediatric intensive care unit in northern Iran; a single center study. J Renal Inj Prev.

[41] Tong A, Hanson CS, Chapman JR, Halleck F, Budde K, Papachristou C (2014). The preferences and perspectives of nephrologists on patients’ access to kidney transplantation: a systematic review. J Transp Secur.

[42] Tong A, Howard K, Wong G, Cass A, Jan S, Irving M (2011). Nephrologists' perspectives on waitlisting and allocation of deceased donor kidneys for transplant. Am J Kidney Dis.

[43] Akolekar D, Oniscu GC, Forsythe JLR (2008). Variations in the assessment practice for renal transplantation across the United Kingdom. Transplantation..

[44] Van Ryn M, Burke J (2000). The effect of patient race and socio-economic status on physicians' perceptions of patients. J Soc Sci Med.

[45] Van Ryn M. Research on the provider contribution to race/ethnicity disparities in medical care. Med Care. 2002;40(1):1140–151. 10.1097/00005650-200201001-00015 11789627

[46] Nelson A (2002). Unequal treatment: confronting racial and ethnic disparities in health care. J Natl Med Assoc.

[47] Green AR, Carney DR, Pallin DJ, Ngo LH, Raymond KL, Iezzoni LI (2007). Implicit bias among physicians and its prediction of thrombolysis decisions for black and white patients. J Gen Intern Med.

[48] Johnson RL, Roter D, Powe NR, Cooper LA (2004). Patient race/ethnicity and quality of patient–physician communication during medical visits. Am J Public Health.

