# Supplementary material for: The global survival rate of graft and patient in kidney transplantation of children: a systematic review and meta-analysis
Source: BMC Pediatr. 2022 Aug 24;22:503. doi: 10.1186/s12887-022-03545-2 (PMC9404642; doi:10.1186/s12887-022-03545-2)
Supplement: Supplementary file 1 — Additional file 1. [file 12887_2022_3545_MOESM1_ESM.docx]

**Table S1**: characteristics of included studies in meta-analysis

| **First author, year of citation** | **country** | **year of publication** | **Time. Period** | **Sample size** | **age donor** | **age recipient** |
| --- | --- | --- | --- | --- | --- | --- |
| Novick, A. C. et. al ,1980([1](#_ENREF_1)) | USA | 1980 | 1968 - 1978 | 58 |  | 6 - 18 years |
| Najarian, J. S. et.al, 1990([2](#_ENREF_2)) | USA | 1990 | 1965-1989 | 75 |  | 6 week - 24 month |
| Ehrich, J. H. et.al, 1991([3](#_ENREF_3)) | Germany | 1991 | 1976- 1989 | 22 |  | 9.8 years ( 7-13 years). |
| Briscoe, D. M. et.al,1992([4](#_ENREF_4)) | USA | 1992 | 1980 - 1990 | 21 | 11 months to 36 years | 1.4 yr (1.1 - two years) |
| Najarian, J. S. et.al, 1992([5](#_ENREF_5)) | USA | 1992 | 1970-1991 | 27 |  | 6 week to 12 month |
| Thomas, G. et.al, 1992([6](#_ENREF_6)) | Ireland | 1992 | 1980-1990 | 38 |  | 8.6 yr(2 to 14 years). |
| Fontaine, E. et.al, 1997([7](#_ENREF_7)) | France | 1997 | 1972 - 1994 | 100 |  | 11.1± 2 4 yr |
| Fontaine, E. et.al, 1998([8](#_ENREF_8)) | France | 1998 | 1972 - 1996 | 14 |  | 12.1 years ( 5 to 18) yr |
| Lashley, D. B. et.al,1999([9](#_ENREF_9)) | USA | 1999 | 1987 - 1997 | 95 |  | 10.9 (1 to 17 years) |
| Chakrabarti, P. et.al, 2000([10](#_ENREF_10)) | USA | 2000 | 1989-1996 | 81 | 27.9±14.5 (0.7–50) | 10.5±5.1 years ( 0.7–17.9) |
| Haberal, M. et.al,2000([11](#_ENREF_11)) | Turkey | 2000 |  | 1286 | 19 to 52 years | 8 to 17 |
| Haberal, M. et.al,2000([12](#_ENREF_12)) | Turkey | 2000 | 1986-1998 | 56 | 39 ±6.7 yr | 14.8±2.3 yr (8±17) |
| Rizvi, S. A. et.al, 2000([13](#_ENREF_13)) | Pakistan | 2000 | 1986-1998 | 52 | 20–65 yrs | 6–15 yrs |
| Adams, J. et.al,2001([14](#_ENREF_14)) | Germany | 2001 | 1977 - 1998 | 30 | 20.94 ±3.03 yr | 4.6 yr (17 months to 6 years) |
| Garcia, C. D. et.al,2001([15](#_ENREF_15)) | Brazil | 2001 | 1989-2000 | 158 | 26 (6- 55) | 3.14 (2-5.9) |
| Khammar, M. A. et.al,2001([16](#_ENREF_16)) | Iran | 2001 | 1989 - 1996 | 41 |  | 6-18yr |
| Samhan, M. et.al, 2001([17](#_ENREF_17)) | Kuwait | 2001 |  | 32 |  | 15.7 (8 - 18) yr |
| Mahdavi, R. et.al, 2002([18](#_ENREF_18)) | Iran | 2002 | 1989-1999 | 49 |  | 12.9 (6-17) years |
| Joshi, S. S. et.al,2002([19](#_ENREF_19)) | India | 2002 |  | 81 |  | 3-16 yr |
| Mehrotra, S.et.al,2002([20](#_ENREF_20)) | India | 2002 | 1984-1996 | 63 | 37.01 ± 8.72 yr | 12.86 ± 2.52 yr |
| Rizvi, S. A. H.et.al,2002([21](#_ENREF_21)) | Pakistan | 2002 | 1986 -1999 | 75 | 39 (20–65 ) yr | 12 ( 6–17 yr) |
| Bartosh, S. M. et.al,2003([22](#_ENREF_22)) | USA | 2003 | 1967-1999 | 217 |  | 12.7 ±4.7(0.4–18 ) yr |
| Englund, M. et.al, 2003([23](#_ENREF_23)) | Sweden | 2003 | 1981- 1991 | 53 |  | median 7.1 years (0.5 yr,15.6 yr) |
| Lufft, V. et.al,2003([24](#_ENREF_24)) | Germany | 2003 | 1983 - 1995 | 40 | 17±14 yr | 10±5 yr |
| Ali-El-Dein, B. et.al,2004([25](#_ENREF_25)) | Egypt | 2004 | 1981-2001 | 195 |  | 13.5 ± 3 (5-18) yr |
| Groothoff, J. W. et.al,2004([26](#_ENREF_26)) | Netherland | 2004 | 1972 - 1992 | 231 |  | <15 yr |
| Hamdi, M. et.al,2004([27](#_ENREF_27)) | Ireland | 2004 | 1989- 2002 | 10 | 30.08 (11-59) yr | 13.4 ( 9–16) yr |
| Ishikawa, N. et.al,2004([28](#_ENREF_28)) | Japan | 2004 | 1983-2002 | 7 |  | <16 yr |
| Jarzembowski, T.et.al,2004([29](#_ENREF_29)) | USA | 2004 | 1995-2002 | 48 |  | 10.9 (1.7–17.8) yr |
| Khositseth, S. et.al,2004([30](#_ENREF_30)) | USA | 2004 | 1986 - 2003 | 20 |  | 9 ± 5 years |
| Kamel, M. H. et.al,2005([31](#_ENREF_31)) | Ireland | 2005 | 1990 - 2003 | 19 | 21.4 ( 10 to 46) yr | 4.7 yr ( 18 months 9 yrs) |
| Mendizabal, S. et.al,2005([32](#_ENREF_32)) | Spain | 2005 | 1979 - 2003 | 15 |  | median 13 yr (6 to 18 years) |
| Otukesh, H.et.al,2005([33](#_ENREF_33)) | Iran | 2005 | 1985 - 2003 | 168 |  |  |
| Otukesh, H.et.al,2006([34](#_ENREF_34)) | Iran | 2006 | 1985-2003 | 278 |  | 11.6 yr |
| Becker, T. et.al,2006([35](#_ENREF_35)) | Germany | 2006 | 1983 -2004 | 442 | Median: 8 ( 1–40) yr | median 2.7 ( 0.9 , 5.9)yr |
| Berber, I.et.al,2006([36](#_ENREF_36)) | Turkey | 2006 | 1991- 2004 | 28 |  | 15.2 ± 2 ( 11 - 17) yr |
| Basiri, A. et.al,2007([37](#_ENREF_37)) | Iran | 2007 | 1985 - 2004 | 86 |  | 11.9 ± 2.77 |
| Basiri, A. et.al,2007([38](#_ENREF_38)) | Iran | 2007 | 1985 - 2004 | 50 | 25.5 ± 3.5 yr | 10.5 ± 4.1 yr |
| Chacko, B. et.al,2007([39](#_ENREF_39)) | India | 2007 | 1991 - 2005 | 90 | 39 ( 22–55) yr | 15 (6 - 18) yr |
| Garcia, C. D. et.al,2007([40](#_ENREF_40)) | Brazil | 2007 | 1989 - 2005 | 38 | 29.9 (19–55) yr | 3.3 ± 1.3 years |
| Rees, L. et.al,2007([41](#_ENREF_41)) | UK | 2007 | 1973 - 2000 | 300 |  | 10.3 ( 1.4–17.9) yr |
| Samhan, M. et.al, 2007([42](#_ENREF_42)) | Kuwait | 2007 | 1993 -2006 | 86 |  | 13.2 (3 - 18) |
| Otukesh, H. et.al,2008([43](#_ENREF_43)) | Iran | 2008 | 1985-2005 | 300 |  | 12.5 ± 2.8 yr |
| Baron, P. W. et.al,2008([44](#_ENREF_44)) | USA | 2008 | 1996 - 2003 | 88 |  | 14.5 ± 4.8 yr |
| Vasudevan, A. et.al,2008([45](#_ENREF_45)) | India | 2008 | 2000 - 2007 | 33 |  | 12.5 ± 3.86 yr |
| Wu, Z. X. et.al,2008([46](#_ENREF_46)) | China | 2008 | 1985 - 2001 | 27 | 27.1 ± 4.8 yr | 12–17 yr |
| Allain-Launay, E. et.al,2009([47](#_ENREF_47)) | France | 2009 | 1995 - 2005 | 553 |  | 9.9 ± 4.4 yr |
| Chavers, B. M. et.al,2009([48](#_ENREF_48)) | USA | 2009 | 2002 - 2005 | 39 |  | 14 ± 3 years ( 7–18 years) |
| Koshy, S. M. et.al,2009([49](#_ENREF_49)) | Canada | 2009 | 1980- 2002 | 274 |  | 10.2 ± 5.0 yr |
| Otukesh, H. et.al,2009([50](#_ENREF_50)) | Iran | 2009 | 1985 - 2006 | 75 |  | 11.86 ± 2.69 yr |
| Oztek, F. Z. et.al,2009([51](#_ENREF_51)) | Austria | 2009 | 1997 - 2005 | 59 |  | 9.6±5.7 yr |
| Wisanuyotin, S. et.al,2009([52](#_ENREF_52)) | Thailand | 2009 | 2001 -2008 | 15 | 25.2 ± 12.9 years (2.9-45). | 12.8 ± 3.2 years ( 5.0-17.6) |
| Benfield, M. R. et.al,2010([53](#_ENREF_53)) | UK | 2010 | 2001- 2004 | 59 |  | 11.36 ±4.89 yr |
| Olaitan, O. K. et.al,2010([54](#_ENREF_54)) | Ireland | 2010 | 1991 - 1997 | 75 | 25.9 (7–53) yr | 12.9 (3–18) yr |
| Otukesh, H. et.al,2010([55](#_ENREF_55)) | Iran | 2010 | 1985 -2005 | 183 | 27 ± 7 yr | 11 ± 3 yr |
| Otukesh, H. et.al,2011([56](#_ENREF_56)) | Iran | 2011 | 1987-2009 | 907 | 27.12 ± 5.96 yr | 13.1 ± 3.54 yr (2-18 yr) |
| Kamal, M. M. et.al,2011([57](#_ENREF_57)) | Germany | 2011 | 1976 - 2009 | 277 | 37.7 ± 8.4 yr | 11.5 ± 3.6 yr |
| Sager, C. et.al,2011([58](#_ENREF_58)) | Argentina | 2011 | 1988 -2007 | 38 |  | 11.89 yr |
| Wafa, E. W. et.al,2011([59](#_ENREF_59)) | Egypt | 2011 | 1976 - 2010 | 273 |  | <17 yr |
| Almasi-Hashiani, A. et.al,2012([60](#_ENREF_60)) | Iran | 2012 | 1999-2009 | 164 | 25.78±13.08 yr | 14±3.16 years |
| Kute, V. B. et.al,2012([61](#_ENREF_61)) | India | 2012 | 1998-2011 | 37 | 38.8 ± 18.6 yr | 13.8 ± 3.1 (7–18) yr |
| Mortazavi, F. et.al,2012([62](#_ENREF_62)) | Iran | 2012 | 1999 - 2009 | 80 |  | 11 ± 3.3 (4–16) year |
| Nehus, E. et.al,2012([63](#_ENREF_63)) | France | 2012 | 2002 - 2009 | 1276 | 27 (19, 38) yr | 13 (8, 16) yr |
| Branco, F. et.al,2013([64](#_ENREF_64)) | Portugal | 2013 | 1984 - 2012 | 124 | 19.7 yr | 13 (10 , 16) yr |
| Hazza, I. et.al,2013([65](#_ENREF_65)) | Jordan | 2013 | 2004 - 2010 | 71 |  | 9.44 ± 2.86 years |
| Huang, P. C. et.al,2013([66](#_ENREF_66)) | Taiwan | 2013 | 1995-2008 | 24 | 40.8 (25- 50) years | 11.6 ± 3.76 (1.75 - 16 )years |
| Sert, I. et.al,2013([67](#_ENREF_67)) | Turkey | 2013 | 1995- 2011 | 61 |  | 14±3 years (4–17 years) |
| Vitola, S. P. et.al,2013([68](#_ENREF_68)) | Brazil | 2013 | 1998 - 2010 | 62 | 30.7 ± 6.8 yr (19–46) | 3.7 ± 2.2 yr (1–12) |
| Shang, W. et.al,2014([69](#_ENREF_69)) | China | 2014 | 2007 - 2013 | 39 | 33.1 ± 16.7(4-59) yr | 13.7 ± 4.0 (4-18) yr |
| Hoseini, R. et.al,2015([70](#_ENREF_70)) | Iran | 2015 | 1985-2012 | 21 |  | 8.3 ±2.5 years ( 2-15 yrs.) |
| Bobanga, I. D. et.al,2015([71](#_ENREF_71)) | USA | 2015 | 2000-2013 | 75 | 37.5 ± 7 yr | 11.7 ± 5.3 yr |
| Papachristou, F. et.al,2016([72](#_ENREF_72)) | Greece | 2016 | 1990 - 2012 | 147 | 44.79 ± 9.55 yr | 2–18 yr |
| Naderi, G. et.al,2017([73](#_ENREF_73)) | Iran | 2017 | 1989-2013 | 297 | 34.7±8.3 (15–53) yr | 11.1±3.7 (3–18) yr |
| Benziane, A. et.al,2017([74](#_ENREF_74)) | Algeria | 2017 | 2007 - 2014 | 32 |  | 12.94 ± 3.66 (5-18 )years |
| Antunes, H. et.al,2018([75](#_ENREF_75)) | Portugal | 2018 | 1981 - 2016 | 101 |  | 13.4 ± 3.2 (3-17) yrs |
| Chavers, B. M. et.al,2018([76](#_ENREF_76)) | USA | 2018 | 1984-2014 | 136 | 31.6 ±9.6 | 1.3 ± 0.4 years |
| Chiodini, B. et.al,2018([77](#_ENREF_77)) | Belgium | 2018 | 1978 - 2016 | 72 | median 23 (7 , 31.2) month | median: 3.2 years (2.3–3.9) |
| El Hennawy, H. M. et.al,2018([78](#_ENREF_78)) | Saudi Arabia | 2018 | 2013 - 2016 | 47 | 30.8±8.82 yr | 10.9 ± 3.79 yr |
| Marlais, M. et.al,2018([79](#_ENREF_79)) | UK | 2018 | 2009-2014 | 46 |  |  |
| Viršilas, E. et.al,2018([80](#_ENREF_80)) | Lithuania | 2018 | 2005 -2015 | 64 |  | 12.85 ± 3.28 years |
| Bulut, I. K. et.al,2019([81](#_ENREF_81)) | Turkey | 2019 | 1998-2018 | 83 | 31 ± 16 yr | 144 ±51 months |
| Bulut, I. K. et.al,2019([82](#_ENREF_82)) | Turkey | 2019 | 1991 - 2018 | 205 | median 30.5 (1- 65) yr | median :11.2 (2-18) yr |
| Cordinhã, C. et.al,2019([83](#_ENREF_83)) | Portugal | 2019 | 1981 - 2016 | 104 | 26.25 ± 12.3 (3 - 59) yrs | 13.7 ± 3.3 ( 3 - 18) years |
| Diaz, J. et.al,2019([84](#_ENREF_84)) | USA | 2019 | 1980 - 2017 | 9 |  | 7.3 ± 5.5 (1.2 - 15) years |
| Kafle, M. P. et.al,2019([85](#_ENREF_85)) | Nepal | 2019 | 2008 - 2018 | 23 | 40.21 ± 8 years | 15.35 ± 1.7 years |
| Kumar, G. et.al,2019([86](#_ENREF_86)) | United Arab Emirates | 2019 | 2010-2018 | 30 |  | 9.8 yr |
| Gholamrezaie, H. et.al,2020 | Iran | 2020 | 2008-2015 | 166 | 27.11 ± 6.45 yr | 12.3 ± 3.3 (3 - 17) years |
| Aoki, Y. et.al,2020 | Japan | 2020 | 1975 - 2009 | 377 | median 39.6 (35.0–44.8) yr | 9.7 ( 1.6–18.8) years |
| Beetz, O. et.al,2020 | Germany | 2020 | 2005 -2018 | 207 | 26.99 (0-64) yr | median: 10.84 (0.3-17.9) years. |

**References:**

1. Novick AC, Karamooz M, Braun WE, Steinmuller D. Results of renal transplantation in children. The Journal of Urology. 1980;124(6):787-9.

2. Najarian JS, Frey DJ, Matas AJ, Gillingham KJ, So S, Cook M, et al. Renal transplantation in infants. Annals of surgery. 1990;212(3):353.

3. Ehrich JH, Brodehl J, Byrd DI, Hossfeld S, Hoyer PF, Leipert K-P, et al. Renal transplantation in 22 children with nephropathic cystinosis. Pediatric nephrology. 1991;5(6):708-14.

4. Briscoe DM, Kim MS, Lillehei C, Eraklis AJ, Levey RH, Harmon WE. Outcome of renal transplantation in children less than two years of age. Kidney international. 1992;42(3):657-62.

5. Najarian JS, Almond PS, Mauer M, Chavers B, Nevins T, Kashtan C, et al. Renal transplantation in the first year of life: the treatment of choice for infants with end-stage renal disease. Journal of the American Society of Nephrology. 1992;2(12):S228.

6. Thomas G, Conlon P, Spencer S, Hickey D, Carmody M, Gill D. Paediatric renal transplantation in ireland: 1980–1990. Irish journal of medical science. 1992;161(8):487-9.

7. Fontaine E, Salomon L, Gagnadoux M-F, Niaudet P, Broyer M, Beurton D. Long-term results of renal transplantation in children with the prune-belly syndrome. The Journal of urology. 1997;158(3):892-4.

8. FONTAINE E, GAGNADOUX M-F, NIAUDET P, BROYER M, BEURTON D. Renal transplantation in children with augmentation cystoplasty: long-term results. The Journal of urology. 1998;159(6):2110-3.

9. Lashley DB, Barry JM, Demattos AM, Lande MB, Mowry JA. Kidney transplantation in children: a single center experience. The Journal of urology. 1999;161(6):1920-5.

10. Chakrabarti P, Wong HY, Scantlebury VP, Jordan ML, Vivas C, Ellis D, et al. Outcome after steroid withdrawal in pediatric renal transplant patients receiving tacrolimus-based immunosuppression. Transplantation. 2000;70(5):760.

11. Sözen H, Dalgic A, Karakayali H, Baskin E, Saatci Ü, Arslan G, et al., editors. Renal transplantation in children. Transplantation proceedings; 2000: Elsevier.

12. Haberal M, Bereket G, Karakayalı H, Arslan G, Moray G, Bilgin N. Pediatric renal transplantation in Turkey: a review of 56 cases from a single center. Pediatric transplantation. 2000;4(4):293-9.

13. Rizvi SAH, Naqvi SA, Hussain Z, Hashmi A, Akhtar F, Hussain M, et al. Renal transplantation in developing countries. Kidney International. 2003;63:S96-S100.

14. Adams J, Güdemann C, Tönshoff B, Mehls O, Wiesel M. Renal transplantation in small children–a comparison between surgical procedures. European urology. 2001;40(5):552-6.

15. Garcia C, Barros V, Schneider L, Guimarães P, Didone E, Guerra E, editors. Renal transplantation in children less than six years old. Transplantation proceedings; 2001.

16. Khammar M-A, Mahdavi-Zafarghandi R, editors. Renal transplantation in children: results of 9 years of renal transplantation in children at our center. Transplantation proceedings; 2001.

17. Samhan M, Al-Mousawi M, Al-Muzairai I, Said T, Ninan V, Nampoory N, editors. Renal transplantation in children. Transplantation proceedings; 2001.

18. Mahdavi R, Naghib M. Kidney transplantation in children: results of ten years exp erience in imam reza hospital. Medical Journal of the Islamic Republic of Iran. 2002;16(3):145-9.

19. Joshi S, Kamat M, Soonawalla F, Bulchand S, Gandhi B, Kaushik V, editors. Paediatric live related renal transplantations: the Jaslok Hospital experience. Transplantation proceedings; 2002.

20. Mehrotra S, Gopalakrishnan G, Chacko K, Kekre N, Abraham B, Gnanaraj L, et al. Paediatric Renal Transplantation—a 15-Year Experience. Asian Journal of Surgery. 2002;25(3):198-202.

21. Rizvi S, Naqvi S, Hussain Z, Hashmi A, Akhtar F, Zafar M, et al. Living‐related pediatric renal transplants: A single‐center experience from a developing country. Pediatric transplantation. 2002;6(2):101-10.

22. Bartosh SM, Leverson G, Robillard D, Sollinger HW. Long-term outcomes in pediatric renal transplant recipients who survive into adulthood. Transplantation. 2003;76(8):1195-200.

23. Englund M, Berg U, Tydén G. A longitudinal study of children who received renal transplants 10–20 years ago1. Transplantation. 2003;76(2):311-8.

24. Lufft V, Tusch G, Offner G, Brunkhorst R. Kidney transplantation in children: impact of young recipient age on graft survival. Nephrology Dialysis Transplantation. 2003;18(10):2141-6.

25. Ali-El-Dein B, Abol-Enein H, El-Husseini A, Osman Y, El-Din AS, Ghoneim M, editors. Renal transplantation in children with abnormal lower urinary tract. Transplantation proceedings; 2004: Elsevier.

26. Groothoff JW, Cransberg K, Offringa M, van de Kar NJ, Lilien MR, Davin JC, et al. Long-term follow-up of renal transplantation in children: a Dutch cohort study. Transplantation. 2004;78(3):453-60.

27. Hamdi M, Mohan P, Little D, Hickey DP. Successful renal transplantation in children with spina bifida: long term single center experience. Pediatric transplantation. 2004;8(2):167-70.

28. Ishikawa N, Tanabe K, Tokumoto T, Ishida H, Miyamoto N, Shinmura H, et al., editors. Transplantation of pediatric cadaveric kidneys into adult or pediatric recipients. Transplantation proceedings; 2004: Elsevier.

29. Jarzembowski T, John E, Panaro F, Heiliczer J, Kraft K, Bogetti D, et al. Impact of non‐compliance on outcome after pediatric kidney transplantation: an analysis in racial subgroups. Pediatric transplantation. 2004;8(4):367-71.

30. Khositseth S, Gillingham KJ, Cook ME, Chavers BM. Urolithiasis after kidney transplantation in pediatric recipients: a single center report. Transplantation. 2004;78(9):1319-23.

31. Kamel MH, Rampersad A, Mohan P, Hickey D, Little D, editors. Cadaveric kidney transplantation in children≤ 20 kg in weight: Long-term single-center experience. Transplantation proceedings; 2005: Elsevier.

32. Mendizabal S, Estornell F, Zamora I, Sabater A, Ibarra FG, Simon J. Renal transplantation in children with severe bladder dysfunction. The Journal of urology. 2005;173(1):226-9.

33. Otukesh H, Sharifian M, Simfroosh N, Basiri A, Hoseini R, Sedigh N, et al., editors. Outcome of renal transplantation in children with low urinary tract abnormality. Transplantation proceedings; 2005: Elsevier.

34. Otukesh H, Basiri A, Simfrosh N, Hoseini R, Sharifian M, Sadigh N, et al. Outcome of pediatric renal transplantation in Labfi Nejad Hospital, Tehran, Iran. Pediatric Nephrology. 2006;21(10):1459-63.

35. Becker T, Neipp M, Reichart B, Pape L, Ehrich J, Klempnauer J, et al. Paediatric kidney transplantation in small children–a single centre experience. Transplant international. 2006;19(3):197-202.

36. Berber I, Tellioglu G, Yigit B, Turkmen F, Titiz M, Altaca G, editors. Pediatric renal transplantation: Clinical analysis of 28 cases. Transplantation proceedings; 2006: Elsevier.

37. Basiri A, Otoukesh H, Simforoosh N, Hosseini R, Farrokhi F. Kidney transplantation in children with augmentation cystoplasty. The Journal of urology. 2007;178(1):274-7.

38. Basiri A, Otukesh H, Hosseini‐Moghaddam S, Ghazi‐Moghaddam B, Haidari M, Sharifian M, et al. Slow graft function after pediatric renal transplantation from volunteer live donors. Pediatric transplantation. 2007;11(5):477-80.

39. Chacko B, Rajamanickam T, Neelakantan N, Tamilarasi V, John GT. Pediatric renal transplantation–a single center experience of 15 yr from India. Pediatric transplantation. 2007;11(8):844-9.

40. Garcia C, Bittencourt V, Pires F, Didone E, Guerra E, Vitola S, et al., editors. Renal transplantation in children younger than 6 years old. Transplantation proceedings; 2007: Elsevier.

41. Rees L, Shroff R, Hutchinson C, Fernando ON, Trompeter RS. Long-term outcome of paediatric renal transplantation: follow-up of 300 children from 1973 to 2000. Nephron Clinical Practice. 2007;105(2):c68-c76.

42. Samhan M, Fathi T, Al-Kandari N, Buresley S, Nampoory MR, Nair P, et al. Renal transplantation in children. Transplantation proceedings. 2007;39(4):911-3.

43. Otukesh H, Basiri A, Simfroosh N, Hoseini R, Fereshtehnejad SM, Chalian M. Kidney transplantation in children with posterior urethral valves. Pediatr Transplant. 2008;12(5):516-9.

44. Baron PW, Ojogho ON, Yorgin P, Sahney S, Cutler D, Ben-Youssef R, et al. Comparison of outcomes with low-dose anti-thymocyte globulin, basiliximab or no induction therapy in pediatric kidney transplant recipients: a retrospective study. Pediatr Transplant. 2008;12(1):32-9.

45. Vasudevan A IA, Jose B, Phadke K. Pediatric renal transplantation: a single-center experience. InTransplantation proceedings 2008 May 1 (Vol. 40, No. 4, pp. 1095-1098). Elsevier.

46. Wu ZX, Yang SL, Wu WZ, Cai JQ, Wang QH, Wang D, et al. The long-term outcomes of pediatric kidney transplantation: a single-centre experience in China. Pediatr Transplant. 2008;12(2):215-8.

47. Allain-Launay E, Roussey-Kesler G, Ranchin B, Guest G, Maisin A, Novo R, et al. Mortality in pediatric renal transplantation: a study of the French pediatric kidney database. Pediatr Transplant. 2009;13(6):725-30.

48. Chavers BM, Chang YC, Gillingham KJ, Matas A. Pediatric kidney transplantation using a novel protocol of rapid (6-day) discontinuation of prednisone: 2-year results. Transplantation. 2009;88(2):237-41.

49. Koshy SM, Guttmann A, Hebert D, Parkes RK, Logan AG. Incidence and risk factors for cardiovascular events and death in pediatric renal transplant patients: a single center long-term outcome study. Pediatr Transplant. 2009;13(8):1027-33.

50. Otukesh H, Hoseini R, Fereshtehnejad SM, Behzadi AH, Chalian M, Jazayeri M, et al. Outcome of living donor renal allograft survival in children with focal segmental glomerulosclerosis. Pediatr Transplant. 2009;13(1):39-43.

51. Oztek FZ, Ipsiroglu O, Mueller T, Aufricht C. Outcome after renal transplantation in children from native and immigrant families in Austria. European journal of pediatrics. 2009;168(1):11-6.

52. Wisanuyotin S, Jiravuttipong A. Pediatric renal transplantation: a single-center experience in northeast Thailand. Journal of the Medical Association of Thailand = Chotmaihet thangphaet. 2009;92(12):1635-9.

53. Benfield MR, Bartosh S, Ikle D, Warshaw B, Bridges N, Morrison Y, et al. A randomized double-blind, placebo controlled trial of steroid withdrawal after pediatric renal transplantation. American journal of transplantation : official journal of the American Society of Transplantation and the American Society of Transplant Surgeons. 2010;10(1):81-8.

54. Olaitan OK, Zimmermann JA, Shields WP, Rodriguez-Navas G, Awan A, Mohan P, et al. Long-term outcome of intensive initial immunosuppression protocol in pediatric deceased donor renal transplantation. Pediatr Transplant. 2010;14(1):87-92.

55. Otukesh H, Hosein R, Fereshtehnejad SM, Riahifard A, Basiri A, Simforoosh N, et al. Short-term and long-term effects of slow graft function on graft survival in pediatric live donor renal transplantation. Pediatr Transplant. 2010;14(2):196-202.

56. Otukesh H, Hoseini R, Rahimzadeh N, Fereshtehnejad SM, Simfroosh N, Basiri A, et al. Outcome of renal transplantation in children: a multi-center national report from Iran. Pediatr Transplant. 2011;15(5):533-8.

57. Kamal MM, El-Hefnawy AS, Soliman S, Shokeir AA, Ghoneim MA. Impact of posterior urethral valves on pediatric renal transplantation: a single-center comparative study of 297 cases. Pediatr Transplant. 2011;15(5):482-7.

58. Sager C, Burek C, Durán V, Corbetta JP, Weller S, Paz E, et al. Outcome of renal transplant in patients with abnormal urinary tract. Pediatric surgery international. 2011;27(4):423-30.

59. Wafa EW, Shokeir AA, Akl A, Hassan N, Fouda MA, El Dahshan K, et al. Effect of donor and recipient variables on the long-term live-donor renal allograft survival in children. Arab journal of urology. 2011;9(2):85-91.

60. Almasi-Hashiani A, Rajaeefard AR, Hassanzade J, Salahi H, Nikeghbalian S, Janghorban P, et al. Graft survival rate of renal transplantation: a single center experience, (1999-2009). Iranian Red Crescent medical journal. 2011;13(6):392-7.

61. Kute VB, Trivedi HL, Vanikar AV, Shah PR, Gumber MR, Patel HV, et al. Long-term outcome of deceased donor renal transplantation in pediatric recipients: a single-center experience from a developing country. Pediatr Transplant. 2012;16(6):651-7.

62. Mortazavi F, Maleki M. Management and outcome of children with end-stage renal disease in northwest Iran. Indian journal of nephrology. 2012;22(2):94-7.

63. Nehus E, Goebel J, Abraham E. Outcomes of steroid-avoidance protocols in pediatric kidney transplant recipients. American journal of transplantation : official journal of the American Society of Transplantation and the American Society of Transplant Surgeons. 2012;12(12):3441-8.

64. Branco F, Almeida F, Cavadas V, Ribeiro S, Osório L, Rocha A, et al. Pediatric kidney transplantation: a single center experience with 134 procedures. Transplantation proceedings. 2013;45(3):1057-9.

65. Hazza I, Al-Mardini R, Salaita G. Pediatric renal transplantation: Jordan's experience. Saudi journal of kidney diseases and transplantation : an official publication of the Saudi Center for Organ Transplantation, Saudi Arabia. 2013;24(1):157-61.

66. Huang PC, Yang CY, Lee CY, Yeh CC, Lai IR, Tsau YK, et al. Pediatric renal transplantation: results and prognostic factors. Asian J Surg. 2013;36(2):53-7.

67. Sert I, Yavascan Ö, Tugmen C, Kara OD, Kilinc S, Dogan SM, et al. A retrospective analysis of long-term graft survival in 61 pediatric renal transplant recipients: a single-center experience. Annals of transplantation. 2013;18:497-504.

68. Vitola SP, Gnatta D, Garcia VD, Garcia CD, Bittencourt VB, Keitel E, et al. Kidney transplantation in children weighing less than 15 kg: extraperitoneal surgical access-experience with 62 cases. Pediatr Transplant. 2013;17(5):445-53.

69. Shang W, Feng G, Gao S, Wang Z, Pang X, Li J, et al. Reduced ATG-F dosage for induction in pediatric renal transplantation: a single-center experience. Pediatr Transplant. 2014;18(3):240-5.

70. Hoseini R, Mirzaiee N, Rahimzadeh N. Renal transplantation outcome in children with cystinosis. International Journal of Children and Adolescents. 2015 Aug 10;1(2):10-2.

71. Bobanga ID, Vogt BA, Woodside KJ, Cote DR, Dell KM, Cunningham RJ, 3rd, et al. Outcome differences between young children and adolescents undergoing kidney transplantation. Journal of pediatric surgery. 2015;50(6):996-9.

72. Papachristou F, Stabouli S, Printza N, Mitsioni A, Stefanidis C, Miserlis G, et al. Long-term outcome of pediatric kidney transplantation: A single-center experience from Greece. Pediatr Transplant. 2016;20(4):500-6.

73. Naderi G, Latif A, Karimi S, Tabassomi F, Esfahani ST. The Long-term Outcome of Pediatric Kidney Transplantation in Iran: Results of a 25-year Single-Center Cohort Study. International journal of organ transplantation medicine. 2017;8(2):85-96.

74. Benziane A, Boutennoune S. Pediatric Kidney Transplant: Experience at an Algerian Nephrology Department. Experimental and clinical transplantation : official journal of the Middle East Society for Organ Transplantation. 2017;15(Suppl 1):97-8.

75. Antunes H, Parada B, Tavares-da-Silva E, Carvalho J, Bastos C, Roseiro A, et al. Pediatric Renal Transplantation: Evaluation of Long-Term Outcomes and Comparison to Adult Population. Transplantation proceedings. 2018;50(5):1264-71.

76. Chavers BM, Rheault MN, Matas AJ, Jackson SC, Cook ME, Nevins TE, et al. Improved Outcomes of Kidney Transplantation in Infants (Age < 2 years): A Single-Center Experience. Transplantation. 2018;102(2):284-90.

77. Chiodini B, Herman J, Lolin K, Adams B, Hennaut E, Lingier P, et al. Outcomes of kidney transplantations in children weighing 15 kilograms or less: a retrospective cohort study. Transplant international : official journal of the European Society for Organ Transplantation. 2018;31(7):720-8.

78. El Hennawy HM, Al Hashemy A, Al Harbi N, Habhab WT, Fahmy AE. A single-center 4-year experience with 47 pediatric renal transplants: Evolving trends. Saudi journal of kidney diseases and transplantation : an official publication of the Saudi Center for Organ Transplantation, Saudi Arabia. 2018;29(6):1303-10.

79. Marlais M, Pankhurst L, Martin K, Mumford L, Tizard EJ, Marks SD. Renal allograft survival rates in kidneys initially declined for paediatric transplantation. Pediatric nephrology (Berlin, Germany). 2018;33(9):1609-16.

80. Viršilas E, Čerkauskienė R, Masalskienė J, Rudaitis Š, Dobilienė D, Jankauskienė A. Renal Replacement Therapy in Children in Lithuania: Challenges, Trends, and Outcomes. Medicina (Kaunas, Lithuania). 2018;54(5).

81. Hebert SA, Swinford RD, Hall DR, Au JK, Bynon JS. Special Considerations in Pediatric Kidney Transplantation. Advances in chronic kidney disease. 2017;24(6):398-404.

82. Bulut IK, Taner S, Keskinoglu A, Toz H, Sarsik B, Sezer TO, et al. Long-Term Follow-up Results of Renal Transplantation in Pediatric Patients With Focal Segmental Glomerulosclerosis: A Single-Center Experience. Transplantation proceedings. 2019;51(4):1064-9.

83. Cordinhã C, Rodrigues L, Carmo C, Gomes C, Macário F, Correia AJ, et al. Pediatric Kidney Transplantation: Experience of a Center Over 4 Decades. Transplantation proceedings. 2019;51(5):1579-84.

84. Diaz J, Chavers B, Chinnakotla S. Outcomes of kidney transplants in pediatric patients with the vertebral defects, anal atresia, cardiac defects, tracheoesophageal fistula, renal anomalies, limb abnormalities association. 2019;23(2):e13341.

85. Kafle MP, Poudyal AK, Chalise PR, Shah DS. Pediatric kidney transplantation in Nepal. Pediatr Transplant. 2019;23(8):e13588.

86. Kumar G, AlIsmaili Z, Ilyas SH, Ayyash BM, Tawfik E, AlMasri O, et al. Good outcome of the single-center pediatric kidney transplant program in Abu Dhabi. Pediatr Transplant. 2019;23(7):e13566.
