# Supplementary material for: The global survival rate of graft and patient in kidney transplantation of children: a systematic review and meta-analysis
Source: BMC Pediatr. 2022 Aug 24;22:503. doi: 10.1186/s12887-022-03545-2 (PMC9404642; doi:10.1186/s12887-022-03545-2)
Supplement: Supplementary file 2 — Additional file 2. [file 12887_2022_3545_MOESM2_ESM.docx]

Table S2: Quality Assessment of included studies

| **Author (year)** | **Selection** | **Comparability** | **Outcome** | **Total** | **Quality*** |
| --- | --- | --- | --- | --- | --- |
| Hashemi (1999) | 2 | 1 | 2 | 5 | Fair |
| Khammar(2001) | 2 | 2 | 1 | 5 | Fair |
| Nafar(2001) | 3 | 2 | 2 | 7 | Good |
| Porooshani(2001) | 2 | 2 | 2 | 6 | Good |
| Shamsa(2001) | 2 | 2 | 2 | 6 | Good |
| Ghods(2002) | 2 | 2 | 1 | 5 | Fair |
| Einollahi(2003) | 2 | 2 | 1 | 5 | Fair |
| Einollahi(2003) | 2 | 2 | 2 | 6 | Good |
| Haghighi(2003) | 2 | 2 | 1 | 5 | Fair |
| Khosroshahi(2003) | 2 | 2 | 2 | 6 | Good |
| Einollahi(2004) | 2 | 2 | 2 | 6 | Good |
| Ghavamzadeh(2004) | 3 | 2 | 2 | 7 | Good |
| Simforoosh(2004) | 2 | 2 | 2 | 6 | Good |
| Ghods(2006) | 2 | 2 | 1 | 5 | Fair |
| Nafar(2006) | 2 | 2 | 1 | 5 | Fair |
| Otukesh(2006) | 2 | 2 | 2 | 6 | Good |
| Roozbeh(2006) | 3 | 1 | 2 | 6 | Good |
| Pourfarziani(2007) | 2 | 2 | 2 | 6 | Good |
| Nazemian(2007) | 3 | 1 | 2 | 6 | Good |
| Nemati(2007) | 2 | 2 | 1 | 5 | Fair |
| Ramezani(2007) | 3 | 1 | 2 | 6 | Good |
| Ramezani(2007) | 3 | 1 | 2 | 6 | Good |
| Yazdani(2007) | 2 | 2 | 1 | 5 | Fair |
| Ataei(2008) | 2 | 2 | 1 | 5 | Fair |
| Ghafari(2008) | 3 | 1 | 2 | 6 | Good |
| Nourbala(2008) | 2 | 2 | 1 | 5 | Fair |
| Shahbazian(2008) | 3 | 2 | 2 | 7 | Good |
| Ashrafi(2009) | 2 | 2 | 2 | 6 | Good |
| Assari(2009) | 2 | 2 | 2 | 6 | Good |
| Ghafari(2009) | 2 | 2 | 1 | 5 | Fair |
| Lankarani(2009) | 2 | 2 | 2 | 6 | Good |
| Naderi(2009) | 3 | 2 | 2 | 7 | Good |
| Mousavi(2010) | 2 | 2 | 1 | 5 | Fair |
| Hashiani(2010) | 2 | 2 | 1 | 5 | Fair |
| Hashiani(2010) | 2 | 2 | 1 | 5 | Fair |
| Hassanzadeh(2010) | 1 | 2 | 2 | 5 | Fair |
| Kakaei(2010) | 3 | 1 | 2 | 6 | Good |
| Hashiani(2010) | 2 | 2 | 1 | 5 | Fair |
| Lankarani(2010) | 3 | 1 | 2 | 6 | Good |
| Moghani-Lankarani(2010) | 2 | 2 | 2 | 6 | Good |
| Taghavi(2010) | 3 | 2 | 2 | 7 | Good |
| Almasi-Hashiani(2011) | 2 | 2 | 2 | 6 | Good |
| Azmandian(2011) | 3 | 1 | 2 | 6 | Good |
| Ghanei(2011) | 2 | 2 | 2 | 6 | Good |
| Hassanzade(2011) | 2 | 2 | 1 | 5 | Fair |
| Najafi(2011) | 2 | 2 | 1 | 5 | Fair |
| Otukesh(2011) | 2 | 2 | 1 | 5 | Fair |
| Akbarzadeh (2012) | 2 | 2 | 2 | 6 | Good |
| Almasi-Hashiani(2012) | 2 | 2 | 1 | 5 | Fair |
| Fattahi(2012) | 2 | 2 | 1 | 5 | Fair |
| Khalkhali(2012) | 3 | 1 | 2 | 6 | Good |
| Mortazavi(2012) | 2 | 2 | 1 | 5 | Fair |
| Soltanian(2013) | 3 | 1 | 2 | 6 | Good |
| Mahdavi Zafarghandi(2013) | 2 | 2 | 1 | 5 | Fair |
| Mahdavi-Zafarghandi(2013) | 3 | 1 | 2 | 6 | Good |
| Rahimzadeh(2013) | 2 | 2 | 1 | 5 | Fair |
| Saatchi(2013) | 2 | 2 | 2 | 6 | Good |
| Farahani(2013) | 2 | 2 | 1 | 5 | Fair |
| Hashemian(2014) | 2 | 2 | 1 | 5 | Fair |
| Mirzaee(2014) | 2 | 2 | 2 | 6 | Good |
| Najafi(2014) | 2 | 2 | 1 | 5 | Fair |
| Rostami(2014) | 3 | 1 | 2 | 6 | Good |
| Rouchi(2014) | 2 | 2 | 2 | 6 | Good |
| Foroushani(2015) | 2 | 2 | 1 | 5 | Fair |
| Khatami(2015) | 2 | 2 | 2 | 6 | Good |
| Mousavi(2015) | 3 | 1 | 2 | 6 | Good |
| Rahimi Foroushani(2015) | 2 | 2 | 1 | 5 | Fair |
| Shahbazi(2015) | 3 | 1 | 2 | 6 | Good |
| Soltanian(2015) | 3 | 1 | 2 | 6 | Good |
| Taghizadeh Afshari(2016) | 2 | 1 | 2 | 5 | Fair |
| Maraghi(2016) | 2 | 2 | 1 | 5 | Fair |
| Monfared(2016) | 3 | 1 | 2 | 6 | Good |
| Shirafkan(2016) | 3 | 2 | 2 | 7 | Good |
| Shirafkan(2016) | 3 | 2 | 2 | 6 | Good |
| Askarishahi(2017) | 3 | 1 | 2 | 6 | Good |
| Ghelichi-Ghojogh(2017) | 2 | 2 | 2 | 6 | Good |
| Rezapour2017) | 2 | 2 | 2 | 6 | Good |
| Ghelichi-Ghojogh(2018) | 2 | 2 | 2 | 6 | Good |
| Rezaei(2019) | 2 | 2 | 2 | 6 | Good |
| Askarishahi(2019) | 2 | 2 | 1 | 5 | Fair |
| Ahmadpour(2020) | 3 | 1 | 2 | 6 | Good |
| Naderi,(2020) | 2 | 2 | 2 | 6 | Good |
| Shahidi(2020) | 3 | 2 | 2 | 7 | Good |
| Shayan(2020) | 3 | 2 | 2 | 7 | Good |
| Shemshadi(2020) | 2 | 2 | 2 | 6 | Good |

* **Newcastle-Ottawa Quality Assessment Form for Cohort Studies**
