# Supplementary material for: The global survival rate of graft and patient in kidney transplantation of children: a systematic review and meta-analysis
Source: BMC Pediatr. 2022 Aug 24;22:503. doi: 10.1186/s12887-022-03545-2 (PMC9404642; doi:10.1186/s12887-022-03545-2)
Supplement: Supplementary file 3 — Additional file 3. [file 12887_2022_3545_MOESM3_ESM.docx]

**Search strategy**

**(Renal Transplantation* OR Kidney Graft* OR renal transplant*) AND (Survival Rate* OR Survival Time* OR Survival Analysis*) AND (Pediatric* OR Child*)**

**(Kidney Transplantation OR Renal Transplantation AND Survival Rate OR Survival Analysis AND Pediatric OR Child AND Iran)**

| **Database** | **Formula** | **No. of Results** |
| --- | --- | --- |
| **PubMed** | ((("Pediatrics"[Mesh]) OR "Child"[Mesh]) AND (("Kidney Transplantation"[Mesh]) AND "Survival Rate"[Mesh])) OR ((Renal Transplantation*[Title/Abstract] OR Kidney Graft*[Title/Abstract] OR renal transplant*)[Title/Abstract] AND (Survival Rate*[Title/Abstract] OR Survival Time*[Title/Abstract] OR Survival Analys*)[Title/Abstract] AND (Pediatric*[Title/Abstract] OR Child*)[Title/Abstract]) | 2985 |
| **Scopus** | ( TITLE-ABS-KEY ( kidney AND transplantation ) OR TITLE-ABS-KEY ( renal AND transplantation ) AND TITLE-ABS-KEY ( survival AND rate ) OR TITLE-ABS-KEY ( survival AND analysis ) AND TITLE-ABS-KEY ( pediatric ) OR TITLE-ABS-KEY ( child ) AND TITLE-ABS-KEY ( Iran ) ) | 1,887 |
| **Web of Science** | **TOPIC**: (((((Kidney Transplantation OR Renal Transplantation) AND (Survival Rate OR Survival Analysis) AND (Pediatric* OR Child*) AND (Iran)))))  Timespan: All years. Indexes: SCI-EXPANDED, SSCI, A&HCI, CPCI-S, CPCI-SSH, BKCI-S, BKCI-SSH, ESCI, CCR-EXPANDED, IC. | 1,276 |
| **ProQuest** | ab((Renal Transplantation* OR Kidney Graft* OR renal transplant*) AND (Survival Rate* OR Survival Time* OR Survival Analys*) AND (Pediatric* OR Child*)) AND (ab(Renal Transplantation* OR Kidney Graft* OR renal transplant*) AND ab(Survival Rate* OR Survival Time* OR Survival Analys*) AND ab(Pediatric* OR Child*)) AND ab((Renal Transplantation* OR Kidney Graft* OR renal transplant*) AND (Survival Rate* OR Survival Time* OR Survival Analys*) AND (Pediatric* OR Child*)) | 34 |
| **Cochrane** | (((((Kidney Transplantation OR Renal Transplantation) AND (Survival Rate OR Survival Analysis) AND (Pediatric* OR Child*) AND (Iran))))) in Title Abstract Keyword - (Word variations have been searched) | 7 |
|  | Duplicates= 1148 after deleting duplicated ref= 4859 |  |
