# Supplementary material for: The global survival rate of graft and patient in kidney transplantation of children: a systematic review and meta-analysis
Source: BMC Pediatr. 2022 Aug 24;22:503. doi: 10.1186/s12887-022-03545-2 (PMC9404642; doi:10.1186/s12887-022-03545-2)
Supplement: Supplementary file 4 — Additional file 4. [file 12887_2022_3545_MOESM4_ESM.docx]

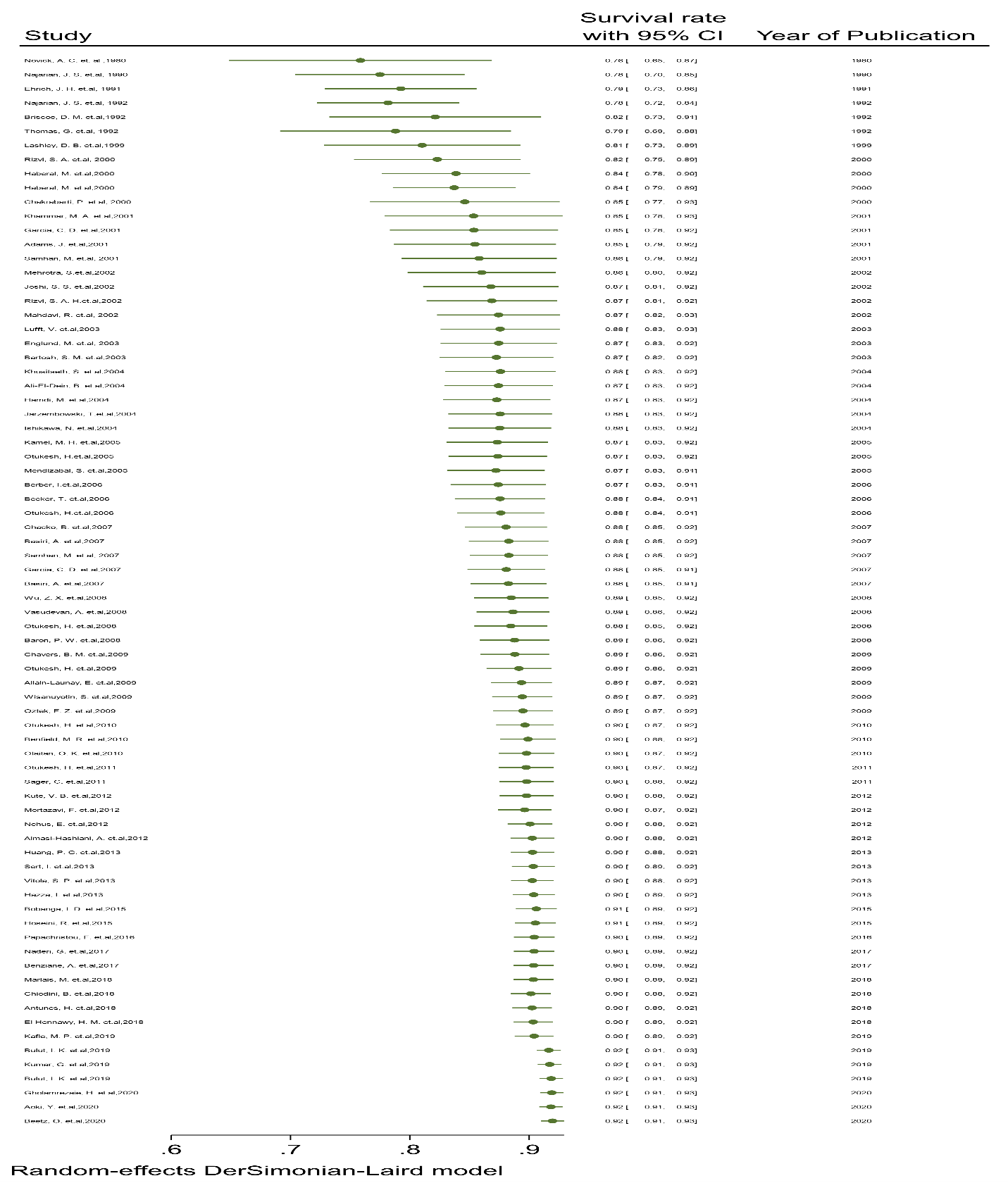


Figure S1. Cumulative meta-analysis of one-year survival of graft kidney transplantation in children


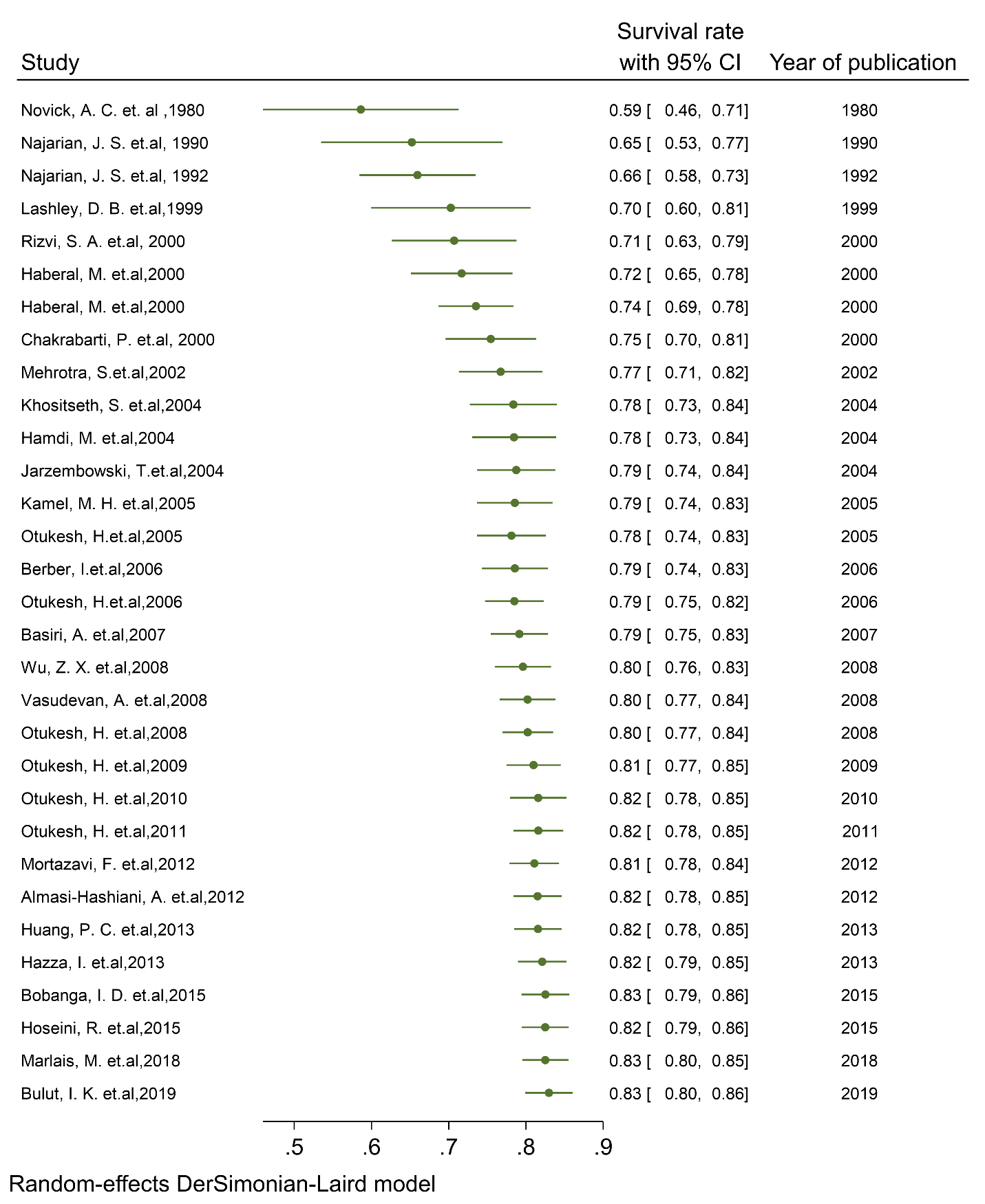


Figure S2. Cumulative meta-analysis of three-year survival of graft kidney transplantation in children


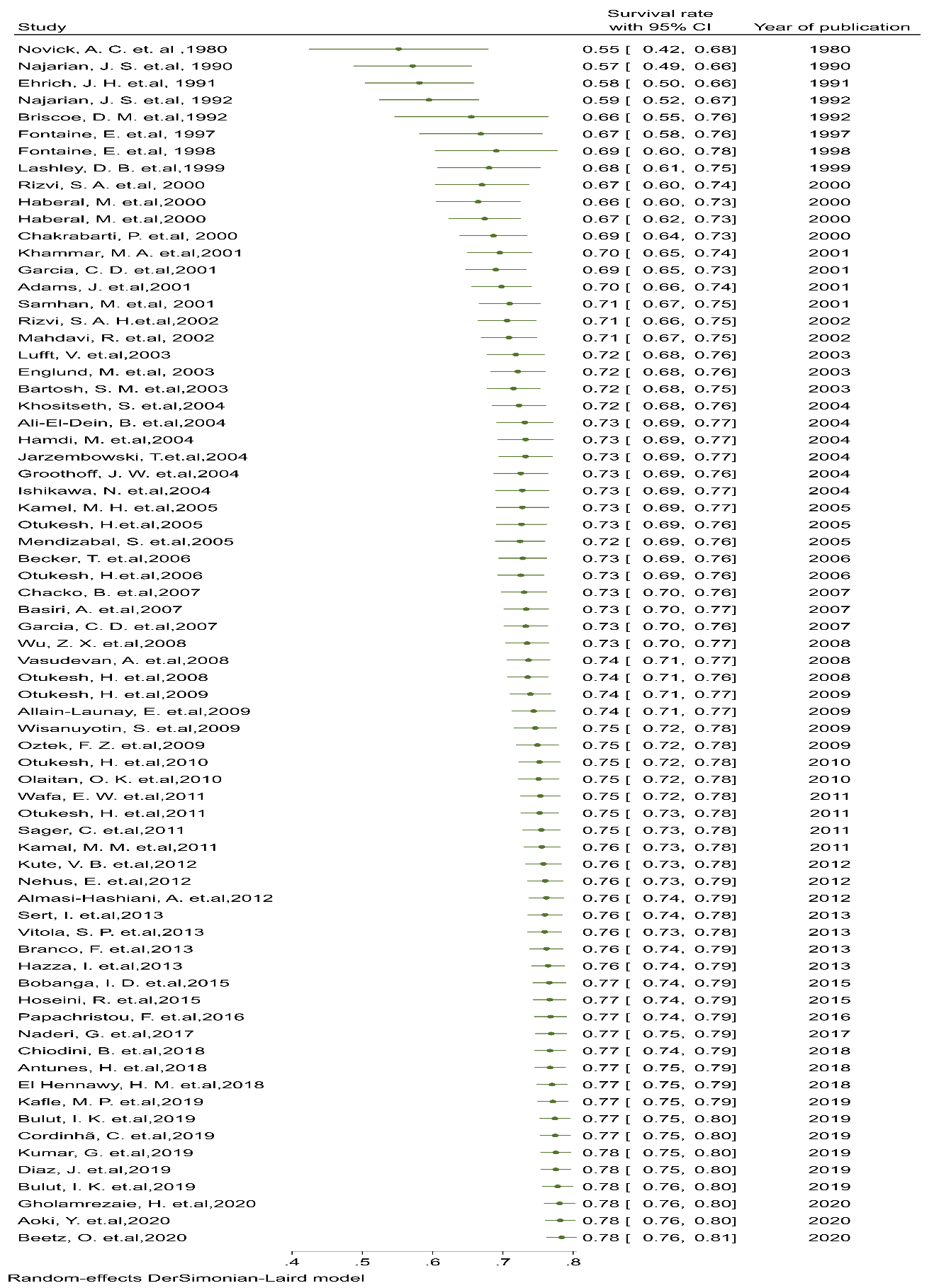


Figure S3. Cumulative meta-analysis of five-year survival of graft kidney transplantation in children


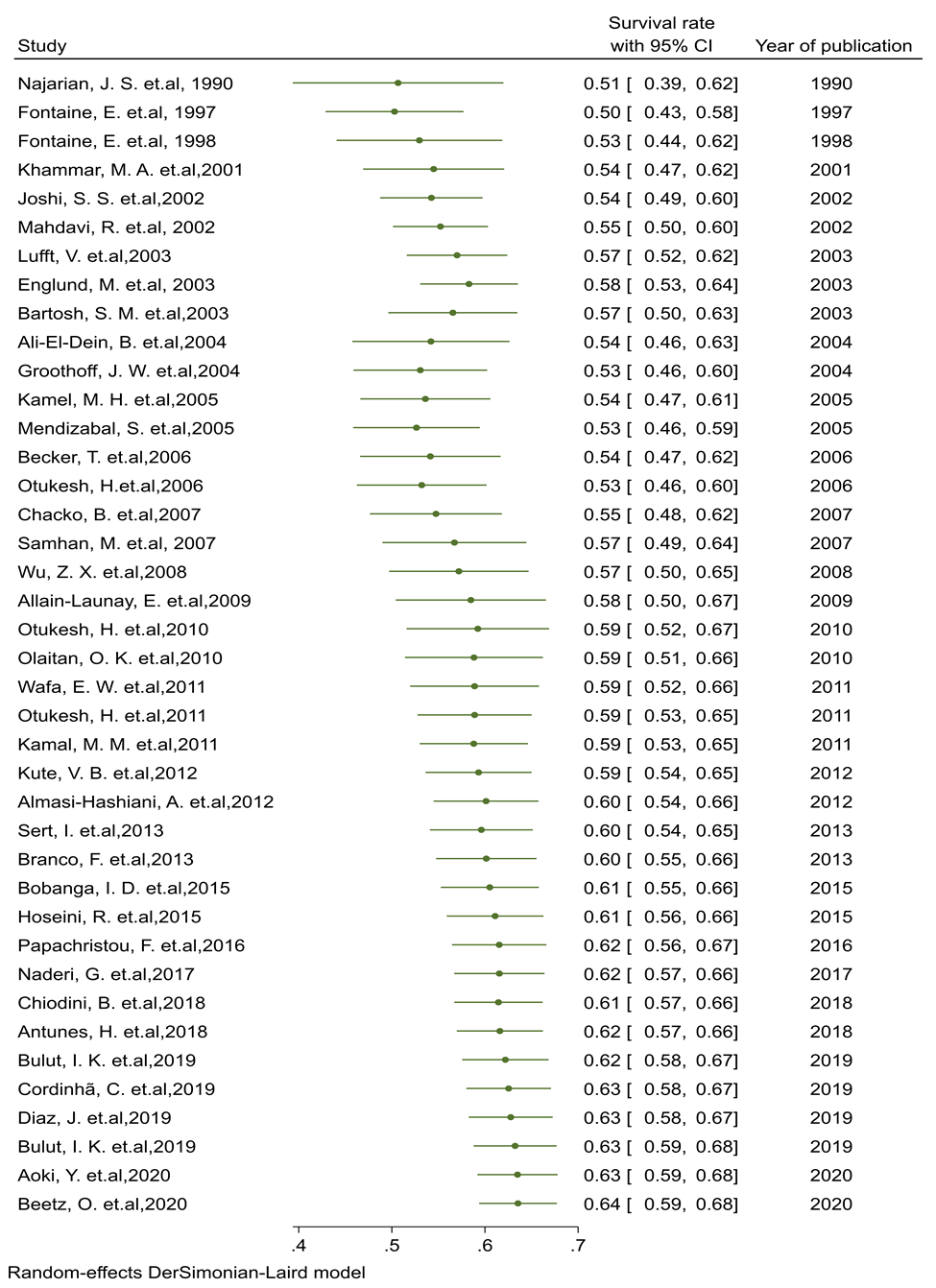


Figure S4. Cumulative meta-analysis of ten-year survival of graft kidney transplantation in children


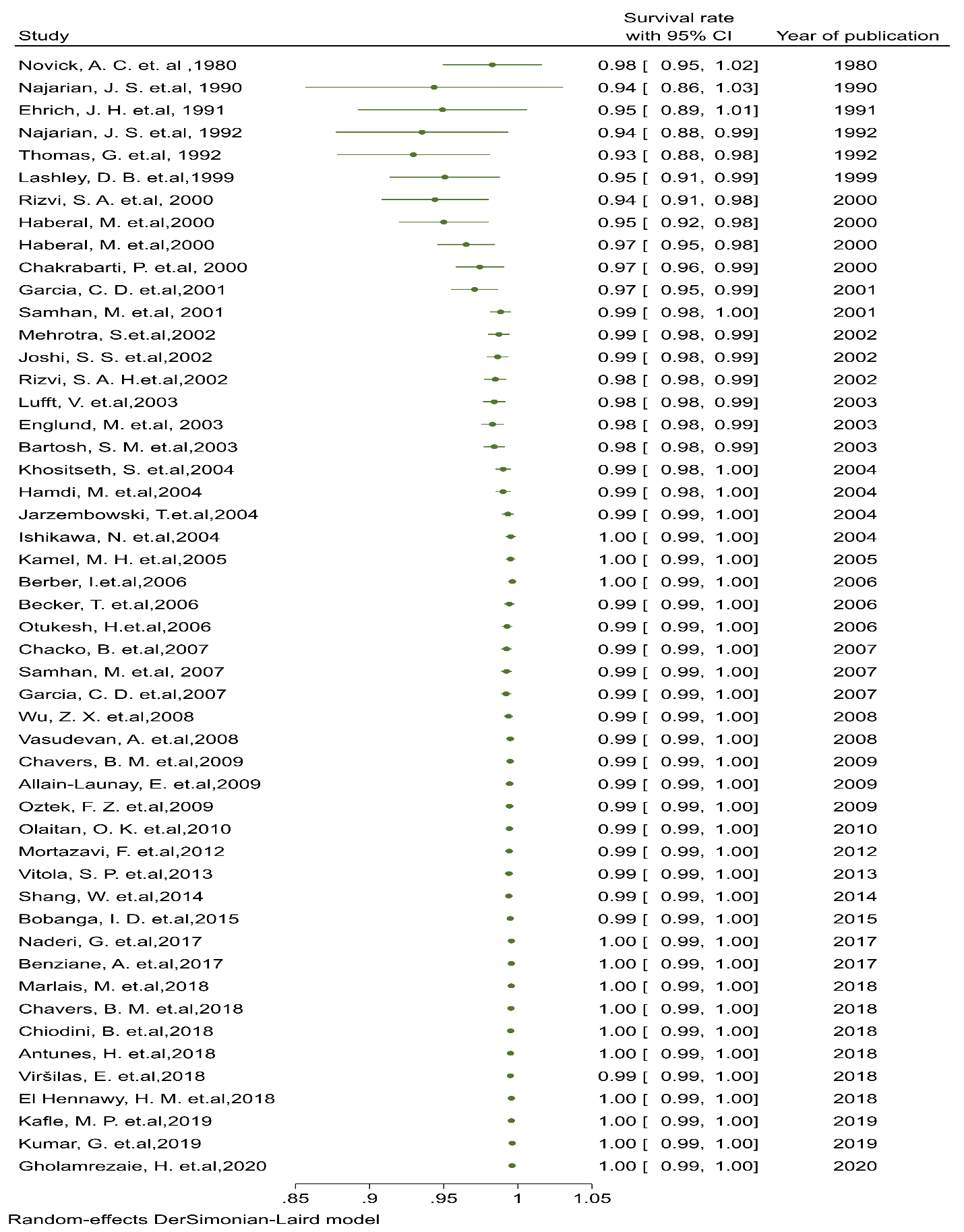


Figure S5. Cumulative meta-analysis of one-year survival of patient kidney transplantation in children


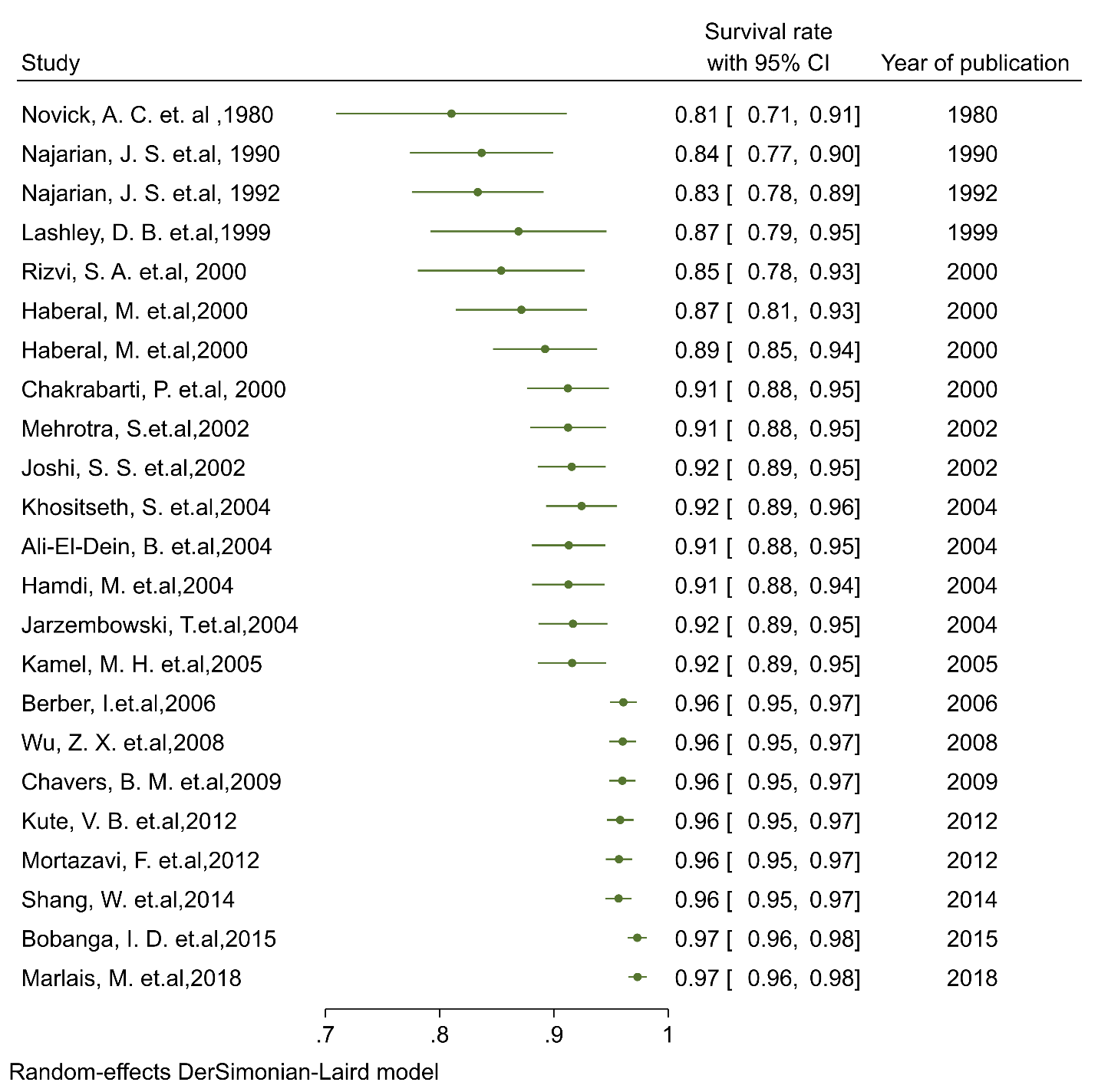


Figure S6. Cumulative meta-analysis of three-year survival of patient kidney transplantation in children


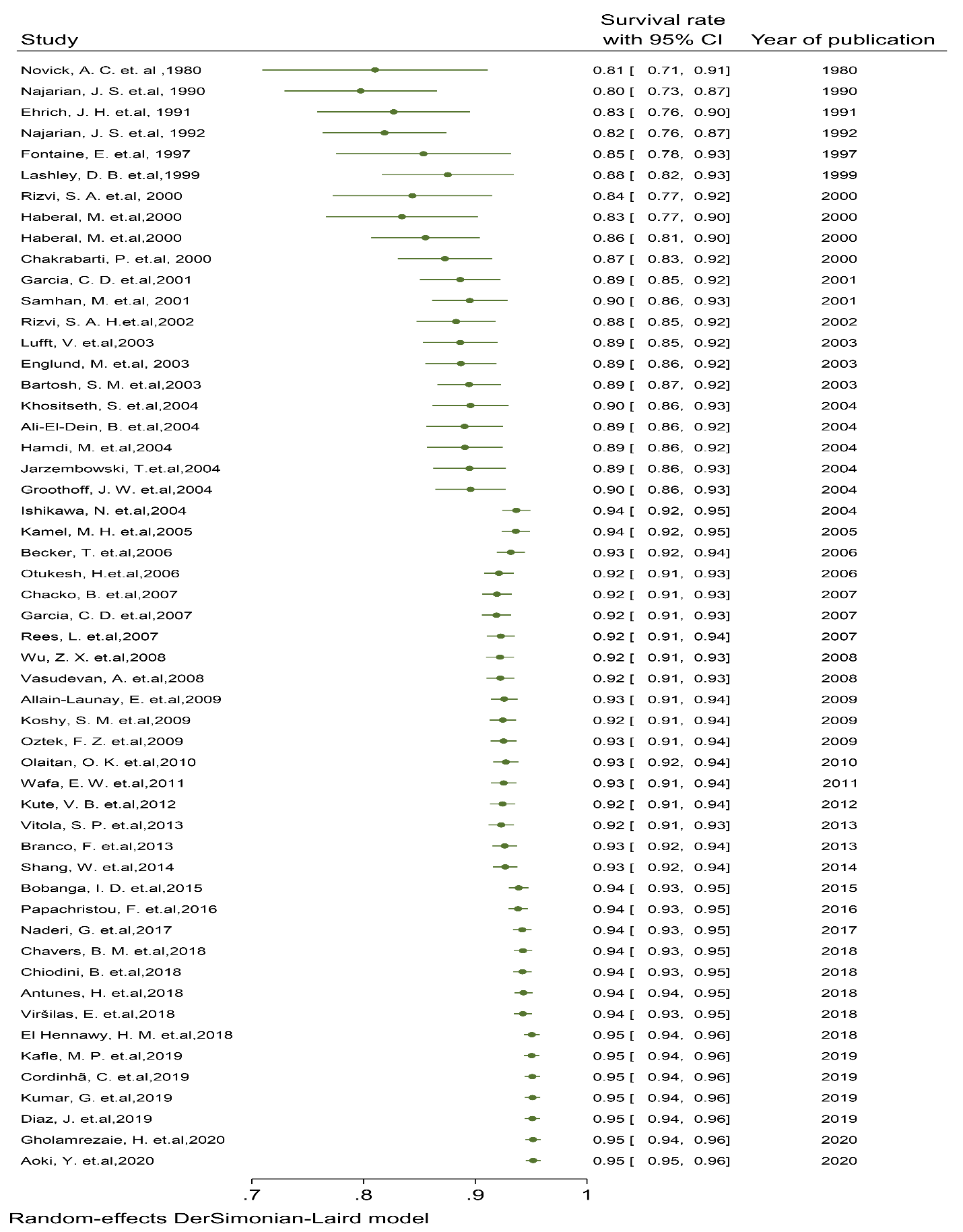


Figure S7. Cumulative meta-analysis of five-year survival of patient kidney transplantation in children
